# Phosphate solubilizing bacteria with glucose dehydrogenase gene for phosphorus uptake and beneficial effects on wheat

**DOI:** 10.1371/journal.pone.0204408

**Published:** 2018-09-21

**Authors:** Muhammad Suleman, Sumera Yasmin, Maria Rasul, Mahreen Yahya, Babar Manzoor Atta, Muhammad Sajjad Mirza

**Affiliations:** 1 Soil and Environmental Biotechnology Division, National Institute for Biotechnology and Genetic Engineering (NIBGE), Faisalabad, Pakistan; 2 Plant Breeding and Genetics Division, Nuclear Institute for Food and Agriculture (NIFA), Peshawar, Pakistan; McGill University, CANADA

## Abstract

The aim of this study was to isolate, characterize and use phosphate solubilizing bacteria to enhance the bioavailability of insoluble Ca-phosphate for wheat plants. For this purpose, 15 phosphorus solubilizing bacteria (PSB) were isolated from wheat rhizospheric soils of Peshawar and southern Punjab region, Pakistan. These isolates were identified using light microscopy and 16S rRNA gene. Among the isolated bacteria, two strains (*Pseudomonas* sp. MS16 and *Enterobacter* sp. MS32) were the efficient P solubilizers based on their P solubilization activity determined qualitatively (solubilization index 3.2–5.8) as well as quantitatively (136–280 μg mL^-1^). These two strains produced indole-3-acetic acid (25.6–28.1 μg mL^-1^), gibberellic acid (2.5–11.8), solubilized zinc compounds (SI 2.8–3.3) and showed nitrogenase and 1-Aminocyclopropane-1-carboxylic acid deaminase activity *in vitro*. Phosphate solubilization activity of *Pseudomonas* sp. MS16 was further validated by amplification, sequencing and phylogenetic analysis of glucose dehydrogenase (*gcd*) gene (LT908484) responsible for P solubilization. Response Surface Methodology for large-scale production was used to find optimal conditions (Temperature 22.5°C, pH 7) for P solubilization. Glucose was found to support higher P solubilization *in vitro*. In an *in vitro* experiment, PSB treated wheat seedlings improved germination and seedling vigor (11% increases) as compared to un-inoculated control. Rhizoscanning of these seedlings showed an increase in various root growth parameters. Wheat inoculation with selected strain MS16 showed pronounced effect on grain yield in pot (38.5% increase) and field (17–18% increase) experiments compared to non-inoculated control. Root colonization by PSB through Florescent *in situ* Hybridization and Confocal Laser Scanning Microscopy confirmed their rhizosphere competence in soil. BOX-PCR confirmed the re-isolated colonies of *Pseudomonas* sp. MS16. The results indicated that gluconic acid producing *Pseudomonas* sp. MS16 from un-explored soils may be cost effective and environment friendly candidate to improve plant growth and phosphorous uptake by wheat plants.

## Introduction

Phosphorus (P) is one of essential chemical elements for all life forms to carry out different metabolic activities as it is an essential component of the most important energy compound ATP and nucleic acids (DNA and RNA) [1]. In plants, phosphorus helps in the process of photosynthesis, proper plant maturation and stress mitigation [2–4]. In soil, phosphorus (P) always forms complexes with other compounds in the form of phosphates [5]. Organic phosphorus in soil is found in plant remains, composts and microbial tissues. Inorganic forms of soil P (Pi) consist of apatite (the original source of all phosphorus), complexed with iron, calcium and aluminum phosphate and phosphorus absorbed onto clay particles. Although, both forms are present in sufficient concentration [6–8], solubility of both organic and inorganic forms in soils is very low. The fixation of Pi into insoluble complexes renders these compounds inaccessible for absorption by plants, and therefore, results in a severe Pi-insufficiency in both acidic and alkaline soils [9]. To address the problem of P deficiency in different crops, phosphatic fertilizers are added in various amounts in soil. These phosphatic fertilizers use rock phosphate as the main source of P_2_O_5_. While the demand continues to increase, rock phosphate supply is limited in the whole world. In addition to low quality of P_2_O_5_ in rock phosphate and high transportation costs, rock phosphate prices are very high [6]. These fertilizers also pose a serious environmental threat, and thus alternate strategies are being developed for sustainable agriculture.

Many studies have reported use of microorganisms to solubilize insoluble phosphate compounds as an alternate strategy to phosphatic fertilizers [10, 11]. Use of microorganisms as bio-fertilizers, on the other hand, has gained much interest in recent era due to their promising effect on growth and yield of plant as well as soil fertility. Bio-fertilizers are also environment friendly and cost effective. Phosphate solubilizing bacteria (PSB) secrete organic acids that dissolve unavailable P (PO_4_^3-^) to plant available forms such as HPO_4_^2-^ and H_2_PO_4_^-^ [12, 13]. During acidification process, the pH may decrease from 7.0 to as low as 2.0 [14–16]. Among acids, gluconic acid is the most frequent organic acid produced by PSB [12, 17]. Gluconic acid is mainly produced in bacteria by an enzyme glucose dehydrogenase in direct oxidation pathway of glucose [18]. This enzyme requires a cofactor Pyrroloquinoline quinone (PQQ) whose biosynthesis involves a PQQ operon consisting of about 5–11 genes [19]. The cloning and expression of genes involved in PQQ biosynthesis demonstrated the importance of the production of gluconic and 2-ketogluconic acid in phosphate solubilization [20]. Chen et al,. have also identified genes for glucose dehydrogenase production [21].

Application of plant growth promoting bacteria (PGPB) as inoculants have shown positive results in increasing grain yield of different crops such as wheat (*Triticum aestivum*), maize (*Zea mays*), rice *(Oryza sativa*) and sugarcane (*Saccharum officinarum)* [22–26]. Phosphate solubilizing bacteria, being an important member of PGPB family, have shown promising results to improve grain yield of cereals and other crops when they were applied as bio-fertilizers [27–29]. Among P solubilizers, *Pseudomonas* and *Bacillus* strains have shown an increase in yield of wheat and other crops due to P-solubilization and organic acid production [30].

Keeping in view the importance of low cost production of wheat using environmentally safe management approach, the present study was designed to isolate P solubilizing bacteria from wheat rhizosphere of different un-explored soils and find their optimal efficacy under different growth and environmental conditions. Additionally, the mechanisms and genes involved in P solubilization were explored.

## Materials and methods

### Sample collection

Rhizospheric soil samples of field-grown wheat were collected from Peshawar (34°00'48.2"N, 71°42'46.5"E) and different districts of southern Punjab i.e. Ali Pur (29°29'12.0"N, 70°54'43.9"E), Bahawalnagar (29°43'37.0"N, 72°57'54.6"E), Bahawalpur (29°22'35.6"N, 71°46'12.2"E), D. G. Khan (30°09'26.8"N, 70°43'48.3"E), Layyah (30°46'02.0"N 70°55'16.2"E), Muzaffargarh (29°56'08.1"N, 71°09'52.8"E), Multan (30°16'00.2"N 71°30'47.3"E) and Rahim Yar Khan (28°40'02.7"N 70°40'13.6"E). No permits were required for collection of plant samples, which complied with all relevant regulations. In addition, the said studies did not involve the endangered or protected species. The samples were brought to Lab and kept at 4°C until further processing for isolation of rhizospheric phosphate solubilizing bacteria.

### Isolation, identification and phylogenetic analysis of phosphate solubilizing bacteria

Phosphate solubilizing bacteria were isolated from rhizospheric samples of wheat varieties at the vegetative stage. Samples of wheat varieties Fakhr-e-Sarhad and Faisalabad-2008 were collected from Peshawar and different districts of southern Punjab, respectively. Phosphate solubilizing bacteria were isolated from wheat rhizospheric soil using serial dilution method [31] on nutrient agar, Pikovskaya agar and National Botanical Research Institute’s Phosphate growth medium (NBRIP)agar plates. Pure bacterial colonies isolated from wheat rhizosphere were further screened for their phosphate solubilizing activity *in vitro* using Pikovskaya agar [32] medium (Yeast extract 0.5 g; dextrose 10.0 g; calcium phosphate 5.0 g; ammonium sulfate 0.5 g; potassium chloride 0.2 g; magnesium sulfate 0.1 g; manganese sulfate 0.0001 g; ferrous sulfate 0.0001 g; agar 15 g; distilled water 1 L; the pH was adjusted to 7.0 ± 0.2 before sterilization) and NBRIP medium (Glucose 10 g; tri-calcium phosphate 5 g; magnesium chloride 5 g; ‎magnesium sulfate (MgSO4.7H_2_O) 0.25 g; potassium chloride 0.20 g; (NH_4_)SO_4_ 0.10 g agar 15 g; distilled water 1 L; the pH was adjusted to 7.0 ± 0.2 before sterilization). [33] containing tri-calcium phosphate as a source of insoluble phosphate. Light microscopy was used to study morphological characteristics, motility and Gram’s reaction of the bacterial isolates [34].

After initial screening, selected PSB were identified on the basis of 16S rRNA gene [35]. For this purpose, DNA from bacterial cultures was extracted using CTAB method. Amplification of 16S rRNA gene was carried out using fD1/rD1 primer pair and profile described by Weisburg et al. [36] (Table 1). Amplified PCR product of ~1.5 Kb length was separated on 1% agarose gel in 1X TAE buffer containing ethidium bromide (20 mg mL^-1^) and was carefully excised from the gel using a sharp blade on UV trans-illuminator. This product was eluted from the gel and cleaned using QIAquick Gel Extraction Kit (QIAGEN Sciences, Maryland 20874, USA) and was commercially sequenced by Macrogen (Seoul, South Korea). The sequences obtained were compared with standard strains in Basic Local Alignment Search Tool (BLAST) in terms of percent identity, query coverage and E value. Sequences were submitted to European Nucleotide Archive (ENA).

**Table 1:** Different PCR primers used in this study.

Genes	Primer sequences (5´-3´)	PCR Profile	Reference
***16S***	F: AGAGTTTGATCCTGGCTCAG R: AAGGAGGTGATCCAGCC	95°C for 5 min, followed by 30 cycles of 1 min at 94°C, 1min at 54°C and 1min at 72°C with final extension at 72°C for 10 min.	[36]
***Gcd***	F: GACCTGTGGGACATGGACGT R: GTCCTTGCCGGT GTAGSTCATC	94°C for 4 min, followed by 35 cycles of 1 min at 95°C, 1min at 56°C and 1min at 72°C with final extension at 72°C for 10 min.	[65]
***pqqD and E***	F: GGCTGCTGGCCGAACTGACTT R: GGCCGCAAGAAGCAT TATTAG	95°C for 5min, followed by 31 cycles of 1 min at 95°C, 1min at 55°C and 1min at 72°C with final extension at 72°C for 10 min.	[20]
***pqqE***	F: GARCTGACYTAYCGCTGYCC R: TSAGSAKRARSGCCTGR	95°C for 1 min, followed by 35 cycles at 94°C for 1 min, at 55°C for 1 min and at 72°C for 2 min, and a final step of 72°C for 10 min.	[66]
***BOX***	F: CTACGGCAAGGCGACGCTGACG	95°C for 1 min, followed by 35 cycles at 94°C for 1 min, at 55°C for 1 min and at 72°C for 2 min, and a final step of 72°C for 10 min	[67]

16S: 16S rRNA was used for phylogenetic analysis, gcd; glucose dehydrogenase gene, pqqE: gene for pqqE biosynthesis, pqqD&E: gene for pqqD&E biosynthesis, BOX: primers used for BOX-PCR analysis

For construction of phylogenetic tree of bacterial strains, closely related sequences were downloaded, aligned and trimmed using MEGA 6. Maximum likelihood (ML) method was used for sequence analysis as mentioned by Mehnaz et al. [24].

### Quantitative estimation of phosphate solubilizing activity

The potential of two phosphate solubilizers i.e. *Pseudomonas* sp. MS16 and *Enterobacter* sp. MS32 to solubilize phosphate was assessed *in vitro* quantitatively initially at 5^th^ day post-inoculation (DPI) only.

Another experiment was setup to study the optimal P solubilization by these strains at 1^st^, 3^rd^, 7^th^, 10^th^ and 15^th^ DPI. Bacterial cultures were grown in triplicate in Pikovskaya broth medium at 30±2°C at 150 rpm on a shaker. Cultures were then centrifuged at 4000 rpm for 10 minutes at 4°C to get cell-free supernatant. Solubilized phosphate in culture supernatant was measured following molybdate blue color method [37] using spectrophotometer (Camspec M350-Double Beam UV-Visible, UK) at 882 nm. To calculate the amount of phosphate, optical density against concentration (μg mL^-1^) of standard solution was plotted on a graph [38]. Standard solution was prepared by dissolving 0.2195 g of KH_2_PO_4_ in 1L water to get 50 μg mL^-1^ solution. Further dilutions (2, 4, 8, 12, 20, 30 μg mL^-1^) were made to get standard curve of KH_2_PO_4_. Un-inoculated sterilized Pikovskaya broth medium was used as control. There were three replicates for each treatment and each treatment received equal amount of culture broth (inoculum adjusted to ~5 x 10^9^ CFU mL^-1^). Change in pH of broth culture in response to P solubilization was recorded for cell-free supernatant measured with a pH meter at each sampling time. The correlation coefficient (r) between soluble P and pH value was calculated using SPSS software (SPSS Inc, Chicago, USA) at 1% level of significance.

### Determination of organic acids in the spent growth medium of PSB

Organic acid profile of PSB strains *Pseudomonas* sp. MS16 and *Enterobacter* sp. MS32 was analyzed through HPLC. For this purpose, cell-free supernatant was filtered using 0.2 μL nylon filter (Millipore, USA). The supernatant (20 μL) was then passed through C-18 column in HPLC (Tubo chrom software, Perkin Elmer, USA) at a flow rate of 0.6 mL min^-1^. Mobile phase used in this case consisted of 30:70 (v/v) Methanol: Water. Organic acid analyzed were gluconic, lactic, malic, acetic, citric, and succinic acid were compared with 100 μg mL^-1^ standards (Sigma). Signals were detected at 210 nm. Peak area and retention time compared with those of standards were used for quantification of organic acids [39].

### Effect of carbon sources on P solubilization activity

To study the effect of carbon source on P solubilizing activity of selected two strains, NBRIP broth medium was supplemented with different carbon sources i.e. Fructose (1%), Glucose (1%), Maltose (1%), Mannitol (1%) and Sucrose (1%). PSB strains MS16 and MS32 were inoculated and kept on a shaker at 30±2°C and 150 rpm for 3 days. Each treatment had three replicates. Sterilized NBRIP medium without inoculation was used as control. Phosphate solubilization activity was measured quantitatively using ammonium molybdate blue color method as described above.

### Optimization of culture conditions using response surface methodology

*Pseudomonas* sp. MS16 solubilized more P using glucose as compared to *Enterobacter* sp. MS32, therefore, MS 16 was selected for further studies using Response Surface Methodology (RSM). RSM was used to optimize the effect of temperature, pH and carbon source i.e. glucose (0.5–15%) on P solubilization. For this purpose, a central composite rotatable design (CCRD) comprising of three factors and four central points was used in this study. The variables used were temperature, pH and carbon source concentration. The Design Expert software (Trial Version 10.0.6; Stat-Ease Inc., USA) was employed to treat the responses. The software provided a set of points as a base design that included both higher and lower values along with some central points. Three dimensional response surface graphs were generated and analyzed using mathematical models [40].

### Amplification of P solubilizing genes

Glucose dehydrogenase (*gcd*) gene was amplified using primers specific for *gcd* gene (Table 1) as described by Chen et al. [21]. Amplification of *pqqE* and *pqqD & E* genes was done using degenerate primers reported by Pérez et al. [41] and Han et al. [20], respectively. Amplified PCR product was separated on 1% agarose gel in 1X TAE buffer containing ethidium bromide (20 mg mL^-1^). DNA ladder (1 Kb) was used as a marker to measure the size of amplified product. The product was excised and was purified using QIAquick Gel Extraction Kit (QIAGEN Sciences, Maryland 20874, USA). Purified PCR product was commercially sequenced by Macrogen (Seoul, South Korea). Sequence scanner software package was used to analyze the sequences and find out their similarity with other sequences in Genbank database using BLAST technique. Sequences were submitted to European Nucleotide Archive. The phylogenetic relationship was studied by neighbor-joining method with help of MEGA6 software [42].

### Bioassays for the detection of plant growth promoting activities of bacterial isolates

*Pseudomonas* sp. MS16 and *Enterobacter* sp. MS32 were screened for plant growth promoting activities. Nitrogen fixation was measured using acetylene reduction assay (ARA) as described by Hardy et al. [43]. Pure bacterial colonies were inoculated to on to NFM (N-free malate) semisolid enrichment medium in vials and incubated at 30±2°C for 48 hours. Following pellicle formation, the bottles were injected with acetylene (10% v/v). After incubation for 16 hours at room temperature, gas samples (100 μL) were analyzed on a gas chromatograph (Thermoquest, Trace G.C, Model K, Rodono Milan, Italy) using a Porapak Q column and a H2-flame ionization detector (FID).

Indole acetic acid (IAA) and gibberellic acid produced was quantified using the protocol as described by Tien et al. [44]. 1-Aminocyclopropane-1-carboxylic acid (ACC) deaminase activity was assessed in vials containing 30 μL of 0.5 M ACC as a sole nitrogen source in 5 mL Dworkin and Foster (DF) minimal salt medium [45].

Zinc solubilization was studied by halo zone formation around bacterial colonies on tris minimal salt medium (Tris Mineral salts medium containing Dextrose10.0g; (NH_4_)_2_SO_4_ 1.0 g, KCl 0.2 g, K_2_HPO_4_ 0.1 g, MgSO_4_-0.2 g; distilled water 1000 ml and pH 7.0 was prepared) containing insoluble salt of zinc i.e. 14 mM ZnO. [46]. A clear zone around a growing bacterial colony indicated zinc solubilization and was measured as zinc solubilization index (SI). SI was calculated as the ratio of the total diameter (colony + halo zone) to the colony diameter [47].

### Biosafety test of PSB

Biosafety test of phosphate solubilizing strains was carried out on blood agar medium. It tests the hemolytic ability of a microbe by forming clear zone in culture plate [48]. For this purpose, commercially available blood agar plates containing 5% (v/v) sheep blood were used. A fresh culture of PSB was streaked onto blood agar plates and incubated at 37±2°C. Hemolysis of red blood cells was observed after 48 h.

### *In planta* evaluation of PSB

#### Plate bioassay to study effect of PSB inoculation on vigor index and root morphology

Seeds of wheat variety “NN-Gandum-1” were surface sterilized with sodium hypochlorite (2%) followed by washing with sterile distilled water. Sterilized seeds were inoculated with *Pseudomonas* sp. MS16 and *Enterobacter* sp. MS32 (1 x 10^9^ CFU mL^-1^), separately and were incubated at 28±2°C in petri dishes (9 cm diameter) containing 0.25% water agar. Un-inoculated seeds dipped in sterilized water were used as control. Different seed germination parameters were studied 7 DPI.

Seedling Vigor index was measured to assess the effect of phosphate solubilizing bacteria on germination of wheat seeds [48]. Germination in seeds is achieved when radicals are half the size of a seed. After 72 hours post-inoculation, radicals were half the size of a seed and percent germination was recorded at that time. The experiment was set up using Completely Randomized Design (CRD). There were three replicates for each treatment and each replicate plate had 21 seeds. Percent (%) germination and seedlings vigor index was calculated using formula described by Islam et al. [49].

Root morphological parameters of wheat seedlings were studied 7 DPI. The experiment was set up in triplicates Three plants from each replicate were collected randomly and different root parameters i.e. Root length, diameter, surface area, volume and number of root tips on each plant were assessed for nine plants through Rhizoscanner (EPSON Perfection V700 Photo, Epson America, Inc. USA), equipped with WinRHIZO software offered by Regent Instruments Co. Canada [50]. The experiment was repeated thrice.

#### Evaluation of PSB for wheat yield parameters

*Pseudomonas* sp. MS16 and *Enterobacter* sp. MS32 were evaluated for their P solubilizing potential in pots with native soil (Loamy, pH 7.5, Organic Matter 0.85%, Available P 1.9 mg Kg^-1^, Total N 0.06%) under net house conditions at NIBGE, Faisalabad (31°23'45.1"N, 73°01'34.2"E) during winter wheat growing season (November 2016—April 2017).

Seeds of wheat variety NN-Gandum-1 were surface sterilized and washed with autoclaved distilled water as described in previous section. Bio-priming of these seeds was carried out by dipping seeds in bacterial cultures 1 x 10^9^ CFU mL^-1^, separately. All the pots were watered once before sowing for proper seed germination. Inoculated seeds were sown in pots with 30 cm diameter containing 12 kg soil (Loamy, pH 7.5 and organic matter 0.85%). There were six plants per pot. Five treatments with six replicates were arranged in CRD. The recommended fertilizer dose for wheat is 150-100-60, N-P-K Kg ha^-1.^ N and P were added in the form of urea 100 Kg hectare^-1^ and Diammonium phosphate (DAP) 50 Kg hectare^-1^, respectively. Fertilizer dose was calculated as recommended one according to pot size and quantity of soil added in individual pot. Treatments with bacterial inoculation were supplemented with 80% of the recommended DAP dose 50 Kg DAP hectare^-1^. Pots un-ammended with 80% DAP and 100% DAP were used as control [51]. All the treatments were supplemented 100% dose of urea. Plants were up-rooted 35 days after sowing and studied for plant growth parameters i.e. plant dry weight, root length and shoot length. Plants were harvested in April 2017 and studied for growth, plant height, number of tillers per plant, plant biomass, grain yield [52] and plant P [51].

Based on promising P solubilization, *Pseudomonas* sp. MS16 was selected for further plant inoculation studies. *Pseudomonas* sp. MS16 (1 x 10^9^ CFU mL^-1^) was tested for its plant growth promoting activity on wheat variety “NN-Gandum-1” in microplots (Loamy soil, pH 7.2, organic matter 0.89%, Available P 1.9 mg Kg^-1^, Total N 0.06%) at NIBGE net house facility over two successive years (2016–17 and 2017–18). Plot size was 1.5 x 1.5 m^2^. There were three replicates microplot for each treatment (100% DAP recommended dose control, 80% DAP recommended dose control and inoculation of *Pseudomonas* sp. MS16 along with 80% of recommended dose of DAP) arranged in randomized complete block design. Sterilized filter mud based on sugarcane waste product (4.3% N, 2% P, 65% organic matter, 6.5–7.5 pH, 3 ms cm^-1^ EC and 40% moisture content, 2 mm particle size) was used as carrier material [53]. Seed pelleting was carried out by mixing the seeds (@ 50 Kg seed in 1 Kg carrier material used for 1 acre) [53, 54] and then left for one hour. Sowing of these seeds was carried out using dibbler in microplots. Plants were provided with N and P as mentioned above for pot experiment. The plants were harvested at maturity and data was recorded for plant height, dry weight, grain weight of plants and P uptake.

*Pseudomonas* sp. MS16 formulated with filter mud as carrier material [53] was used for wheat varieties Fakhr-e-Sarhad and NN-Gandum-1 under field conditions during winter season of 2016–17 at Nuclear Institute for Food and Agriculture (NIFA) (34°00'48.2"N, 71°42'46.5"E), Peshawar and NIBGE, Faisalabad (31°23'44.0"N, 73°01'37.2"E), respectively (S1 Table). Experiment was conducted in random complete block design. Each treatment consisted of four replicates and a plot size 7m x 4m and 6m x 6m at NIFA, Peshawar and NIBGE, Faisalabad, respectively. Sowing of seeds was carried out by drill sowing method using a hand drill. The experiment was conducted with the same doses of fertilizers, treatments, controls and agronomic practices as used in net house experiment.

### Effect of PSB inoculation on P uptake of wheat and soil properties

#### Estimation of phosphorus in plant samples

Phosphorus contents were determined in fully matured wheat plants grown in pots, micro plots and fields. Seed P contents were estimated for all three treatments i.e. PSB inoculation with 80% DAP, 100% control (100% DAP), 80% Control (80% DAP).

Ground seed material (1g) per plant was selected for plant P analysis. Dried ground seed samples were subjected to tri-acid digestion as described by Gorsuch et al. Total P of plant seed material was measured as described by Olsen et al. [55]. There were six replicates for pot experiment and three replicates for micro plots and field experiments.

#### Estimation of phosphorus, sodium, potassium and phosphatase activity in soil samples

Rhizospheric soil from each experimental site and pots collected at harvesting stage was used to evaluate the effect of inoculated bacteria on soil characteristics. Soil sample was air dried and subjected to digestion by perchloric acid. Samples were filtered after digestion and filtrate was analyzed for available phosphorus. Exchangeable K and sodium content was determined using a flame photometer following the method described by Simard et al. [56–58]. Soil phosphatase activity was assayed using *p*-nitrophenyl as substrate [59].

### Detection and colonization of inoculated PSB

The plants of wheat variety NN-Gandum-1 grown in pots under net house conditions were also studied for viability and colonization of inoculated phosphate solubilizing bacteria was detected from the rhizosphere using viable count [60], BOX-PCR [61] and fluorescent *in situ* hybridization (FISH) [62].

Viable count method was used to monitor changes in population of inoculated strains (*Pseudomonas* sp. MS16 and *Enterobacter* sp. MS32) associated with plant roots. The isolated colonies were identified on the basis of morphological characters along with other characteristics i.e. P solubilization, IAA production and Zinc solubilization [63].

BOX-PCR was used to confirm the presence of inoculated *Pseudomonas* sp. MS16 by expression of strain specific banding pattern. For this purpose primer BOX-A1R was used (Table 1) [64]. Amplified PCR product was separated on 1% agarose gel in 1X TAE buffer containing ethidium bromide. 1kb and 100bp DNA ladders (Fermentas, Germany) was used as a size markers. The gel was visualized under UV light using gel documentation system (Vilbour Lourmat CN-1000-26MX, France). BOX-PCR fingerprints of the bacterial colonies re-isolated by viable count were compared with those of the pure culture of the inoculated *Pseudomonas* sp. MS16 [61].

Colonization of *Pseudomonas* sp. MS16 and *Enterobacter* sp. MS32 was studied using FISH. Fluorescently labelled oligonucleotide probes EUB338 Mix was labelled with fluorescein while PSM and GAM42a were labelled with Cy3 at 5´ end (Interactiva Biotechnologie GmbH, Ulm, Germany). The experiment was performed following the method as described by Amann et al. [62]. Plants were carefully removed 21 DPI of the selected strains and the roots were washed with sterilized PBS. Fixation buffer (4% paraformaldehyde in PBS) was used to fix cells on the surface of roots. Samples were incubated for 1h at 4°C. The samples were washed with 50, 80 and 96% Ethanol for 3 minutes each and were kept at room temperature. For hybridization, the root samples were fixed on a glass slide and treated with hybridization buffer (30 μL) and 15 pmol of each probe. FLUOS-labelled EUB 338 Mix specific for bacteria was used for all samples. Cy3-labelled probes, PSM specific for *Pseudomonas* spp. and GAM42a specific for *gamma Proteobacteria* were used for *Pseudomonas* sp. MS16 and *Enterobacter* sp. MS32, respectively. Hybridization was carried out at 46°C for 3 h. Root samples were washed in pre-warmed washing buffer for 20 minutes. After this, roots were washed with sterilized water, air-dried and mounted on a microscopic slide in citi-fluor. The slides were observed using confocal laser scanning microscope (CLSM) (Olympus, FV 1000, Japan) equipped with FV10-ASW 1.7 software tool and argon laser for excitation of FLOUS at 488 nm and Cy3 at 514 nm.

Image J software tool was used to analyze the images obtained from CLSM [68]. The cell count in images was calculated using “Analyze Particle” feature in the software. Upper and lower threshold was optimized to eliminate the non-specific objects [69].

### Statistical analysis

Regression and correlation analysis was done on different P solubilizing parameters i.e. change in pH and P solubilization using SPSS software Package Version 23.0 (SPSS Inc., USA). Lab and field experiments data was statistically analyzed by ANOVA and difference between the treatments was compared by least significant difference (LSD) test at 1% and 5% level of confidence respectively using STATIX 10.0 software (Tallahassee, FL, USA). Regression and Response surface graph analysis was performed for effect of pH, temperature and concentration of carbon source using Design Expert software (State Ease, USA).

## Results

### Isolation, screening and identification of rhizospheric phosphate solubilizing bacteria

**Fig 1:**
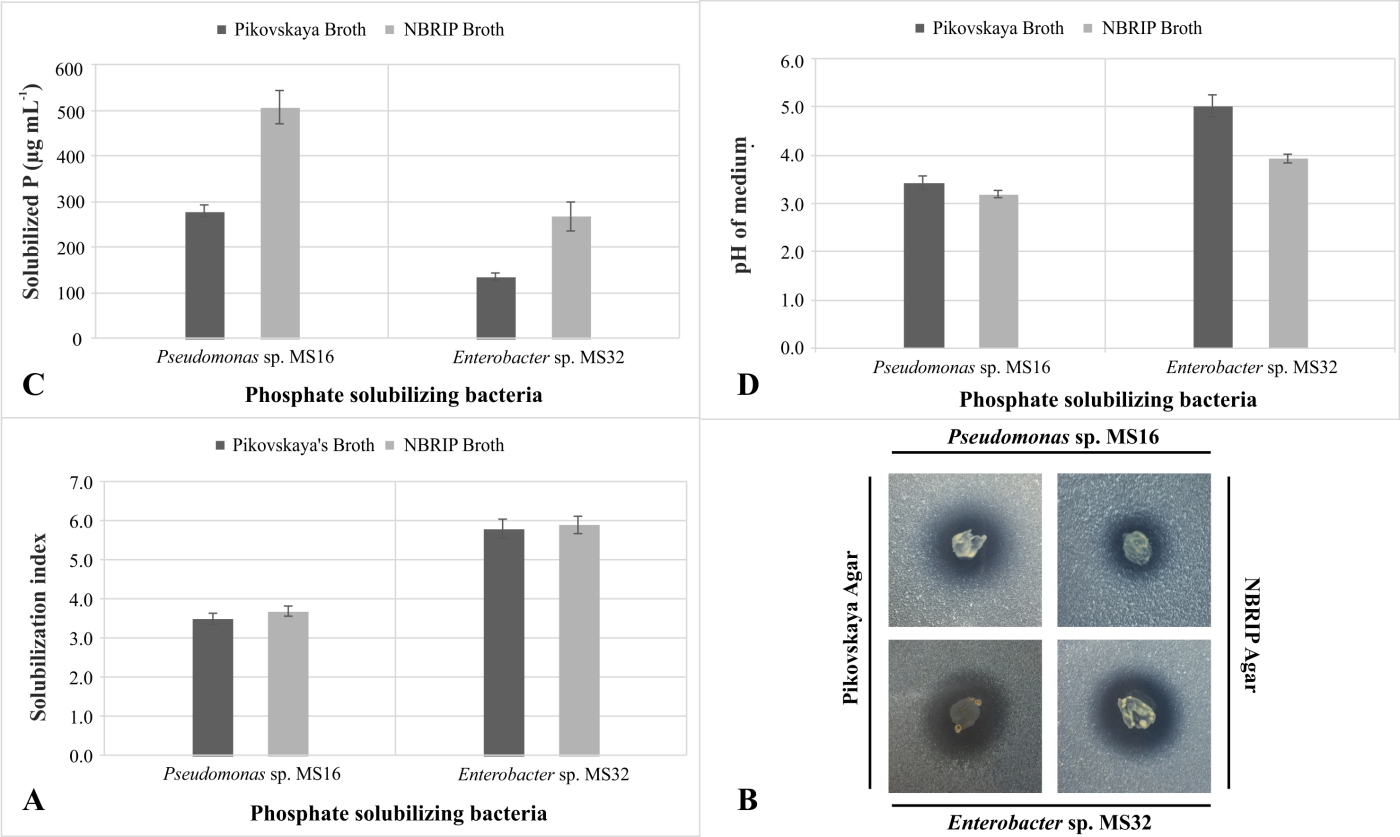
*In vitro* P solubilization activity of wheat rhizosphere associated bacteria. A: Solubilization Index of PSB on two different media, B: Halo zone formation by PSB on two different media, C: Quantitative estimation of phosphate solubilized in two different media, D: Change in pH of medium during P solubilization by PSB at 5 DPI.

From wheat rhizospheric soil samples collected from Peshawar and different districts of southern Punjab, 170 phenotypically different bacterial colonies were obtained. Only 15 colonies showed phosphate solubilization activity as indicated by halo zone formation on Pikovskaya agar and NBRIP agar plates (S1 Fig). In qualitative assays, solubilization index by fifteen selected PSBs in Pikovskaya agar medium ranged between 2.2–5.8 while it ranged between 2–15 mm in NBRIP medium. Two bacterial strains i.e. *Pseudomonas* sp. MS16 and *Enterobacter* sp. MS32 showed maximum phosphate solubilization activity. Higher zone of solubilization (Fig 1) in both media (Pikovskaya and NBRIP) was observed by MS32 (SI: 5.8 in Pikovskaya). Light microscopy of selected bacterial isolates MS16 and MS32 showed that cells were motile, rod shaped and Gram negative (Table 2).

**Table 2:** Morphology, identifition and characterization of P solubilizing bacteria for growth promoting traits.

Strain	^1^Identification	Accession no.	^2^Colony Morphology	^2^Cell Morphology	^3^IAA Production (μg mL^-1^)	^4^Gibberellic acid Production (μg mL^-1^)	^4^ARA Activity (nmol ethylene h^-1^ mg protein^-1^)	^5^Zinc Solubilization (SI)	^6^ACC Deaminase Activity
MS16	*Pseudomonas koreensis*	LT908012	Yellow, circular, entire margins, shiny, convex	Long rods, motile	25.6±1.40	2.5±0.70	8±0.2	2.8±0.18	++
MS32	*Enterobacter cloacae*	LT908013	Off-white, circular, entire margins, shiny, convex	Small rods, motile	28.1±1.23	11.8±3.11	19±0.3	3.3±0.12	+++

^1^Bacteria were identified on the basis of 16S rRNA gene amplification and sequencing

^2^Colony and cell morphology of pure bacterial cultures were studied using light microscopy at 100X.

^3^Indole acetic acid (IAA) was quantified by HPLC.

^4^Acetylene Reduction Assay (ARA) activity quantified by GC.

^5^Zinc Solubilization detected by plate assay, Solubilization index (SI) on Pikoviskaya agar

^6^1-Aminocyclopropane-1-carboxylic acid (ACC) deaminase activity was qualitatively studied by using ACC as a sole nitrogen source in DF minimal medium. + represents level of growth

**Fig 2:**
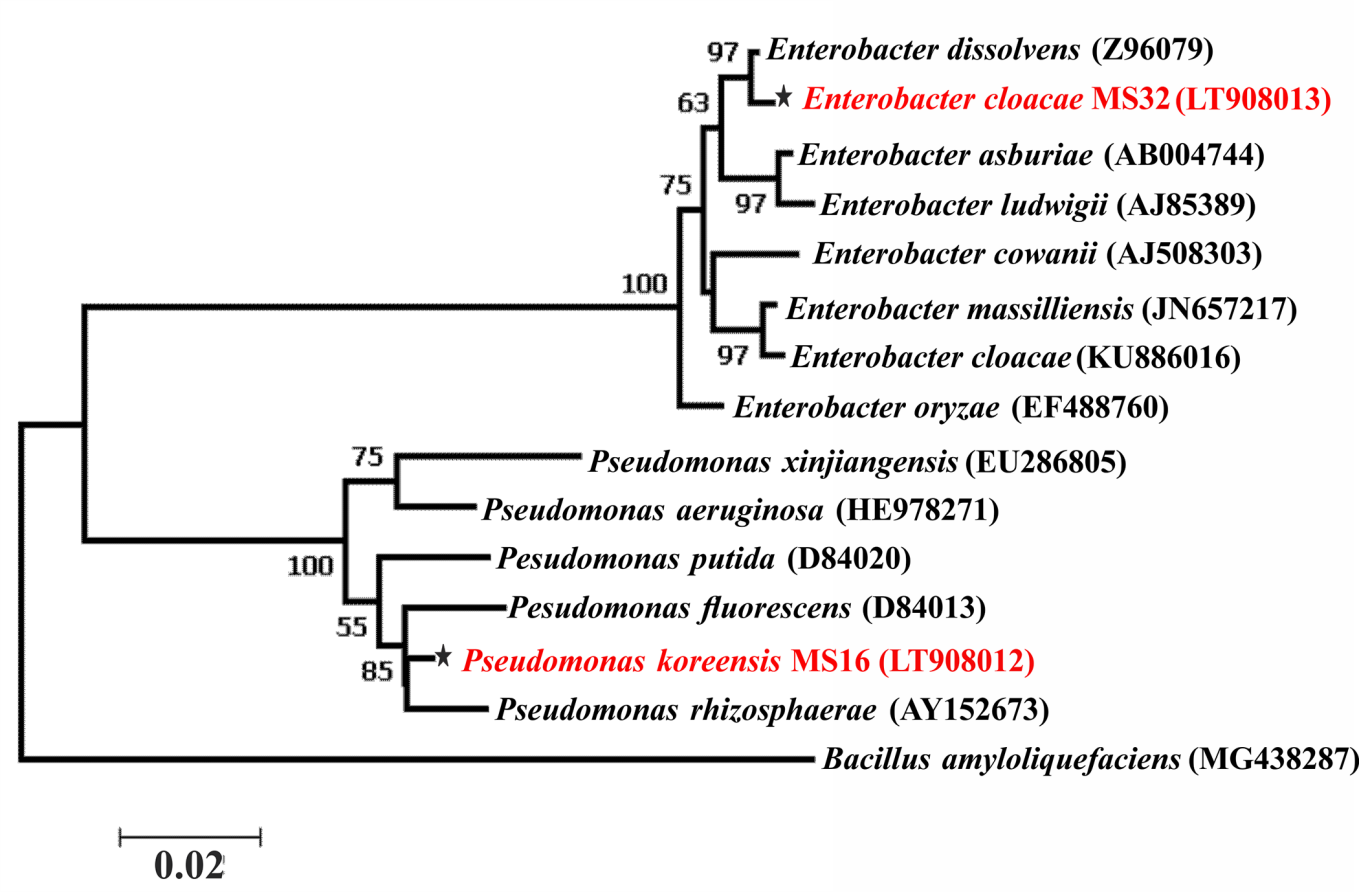
16S rRNA sequence-based phylogenetic tree of PSB strains isolated from wheat rhizosphere, Phylogenetic analysis of PSB strains was done by aligning sequences with already reported type strains using CLUSTALW method. Tree was generated using maximum likelihood method.

P solubilizing bacteria were identified based on PCR amplification of 16S rRNA gene. Sequence analysis of 16S rRNA gene was done using NCBI BLAST tool based on % homology, E-Value, and query coverage (S2 Table). 16S rRNA gene sequencing of strains MS16 and MS32 showed 99% sequence homology with *Pseudomonas koreensis* and *Enterobacter cloacae*, respectively. 16S rRNA gene sequences of *Pseudomonas* sp. and *Enterobacter* sp. were allocated with NCBI accession numbers LT908012 and LT908013, respectively (Table 2). Phylogenetic tree was constructed based on Maximum likelihood method (Fig 2). PSB strains MS16 and MS32 were submitted to NIBGE Biotech Resource Centre (NBRC), Pakistan and allocated with accession number NBRC419 and NBRC421, respectively.

### Quantitative estimation of phosphate solubilizing activity

In quantitative assay, *Pseudomonas* sp. MS16 showed maximum P solubilization activity (280 μg mL^-1^) followed by *Enterobacter* sp. MS32 (136 μg mL^-1^) at 5 DPI. Maximum decrease in pH was observed in culture filtrate inoculated with *Pseudomonas* sp. MS16 (initial pH at 7 to 3.58) (Fig 1).

**Fig 3:**
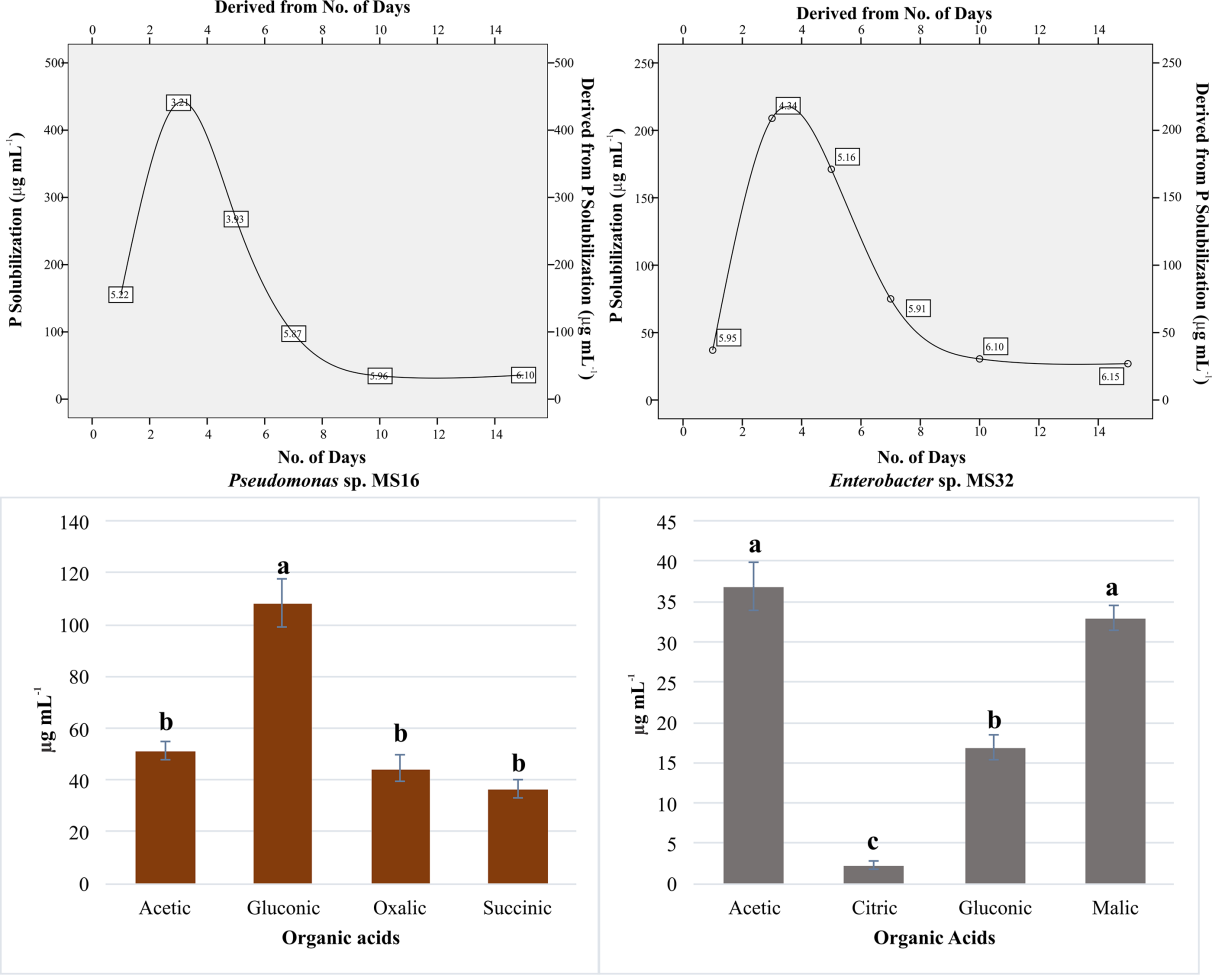
Interaction between phosphate solubilization and pH at different time intervals and organic acid production by PSB Phosphate solubilization by PSB was assessed on 1^st^, 3^rd^, 7^th^, 10^th^ and 15^th^ DPI. Un-inoculated sterilized Pikovskaya’s broth medium was used as control. Each treatment received equal amount of culture broth (~5 x 10^9^ CFU mL^-1^). Organic acids produced by phosphate solubilizing bacteria in liquid medium during P solubilization were quantified using HPLC. Acetic, citric, gluconic, gibberellic, malic, oxalic and succinic acid were detected. Means are an average of three replicates. Means followed by different letter indicates significant difference (P<0.01) in data for that strain.

In another experiment, P solubilization activity of both these strains was measured at different time intervals. P solubilization gradually increased from 24 to 72 h, and decreased thereafter (at 96 h) in the culture-filtrates of both the strains (Fig 3). The concentration of solubilized phosphate varied from ~27 μg mL^-1^ to ~440 μg mL^-1^ in the culture filtrates of these strains. Maximum P solubilization, observed in case of *Pseudomonas* sp. MS16 and *Enterobacter* sp. MS32, was ~440 μg mL^-1^ and ~209 μg mL^-1^, respectively. Lowest P solubilization by *Enterobacter* sp. MS32 was found at 15^th^ DPI (27 μg mL^-1^). pH decreased from 7 at the time of inoculation to as low as 4.19 and 4.34 in case of *Pseudomonas* sp. MS16 and *Enterobacter* sp. MS32, respectively at 3^rd^ DPI (Fig 3).

### Determination of organic acids in the spent growth medium of PSB

PSB were studied for organic acid production using HPLC. *Pseudomonas* sp. MS16 produced gluconic acid (102 μg mL^-1^), acetic acid (54 μg mL^-1^) and oxalic acid (48 μg mL^-1^). Prominent organic acids produced by *Enterobacter* sp. MS32 were acetic acid (34.8 μg mL^-1^) and malic acid (31.8 μg mL^-1^) (Fig 3).

### Effect of carbon sources on P solubilization activity

**Fig 4:**
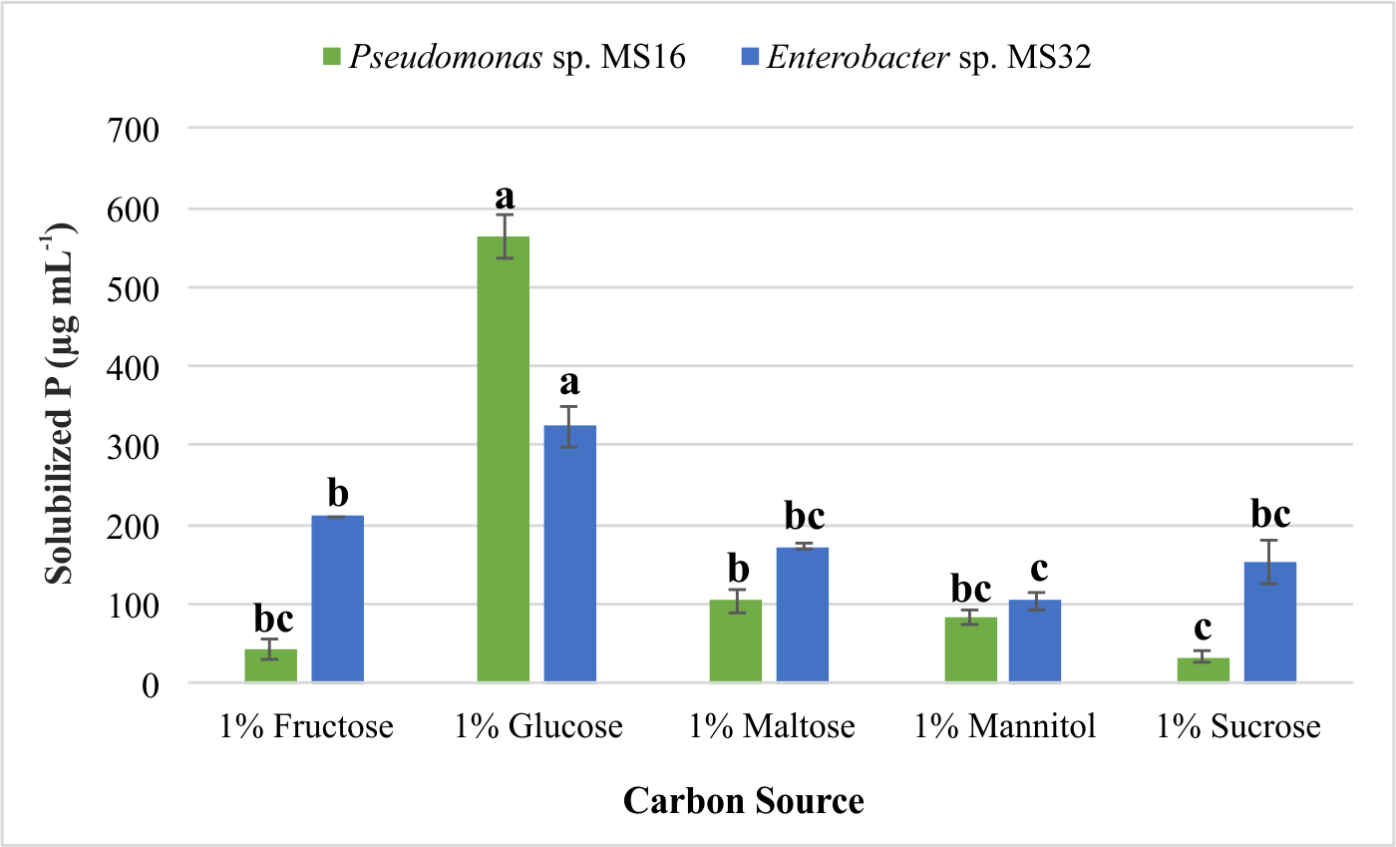
Effect of different carbon sources on phosphate solubilization. NBRIP broth medium supplemented with different carbon sources was inoculated with PSB and kept on a shaker at 30±2°C and 150 rpm for 3 days. Each treatment had three replicates. Sterilized NBRIP medium without inoculation was used as control. Phosphate solubilization activity was measured quantitatively using ammonium molybdate blue color method.

NBRIP medium supplemented with different carbon sources was used to evaluate P solubilization activity of selected isolates. P solubilization was maximum when glucose was supplied as a carbon source to both *Pseudomonas* sp. MS16 and *Enterobacter* sp. MS32 (Fig 4). *Pseudomonas* sp. MS16 solubilized more P (~550 μg mL^-1^) using glucose as compared to *Enterobacte*r sp. MS32 (~323 μg mL^-1^).

### Optimization of culture conditions using response surface methodology

RSM plot showed that phosphate solubilization increased with temperature (22.5°C) and pH (7.0) up to an optimum point where further increase in temperature or pH resulted in decrease of P solubilization activity. Carbon source (glucose) concentration had a positive correlation with phosphate solubilization. Phosphate solubilization by *Pseudomonas* sp. MS16 was found to be maximum (472 μg mL^-1^) at 22.5°C incubation temperature, initial pH of medium at 7.0 and 1.5% carbon source (glucose) concentration in NBRIP medium after incubation for 3 days. When analyzed separately, maximum P solubilization (495 μg mL^-1^) was obtained at a temperature ranging between 22.5°C-25°C. Minimum phosphate was solubilized (103 μg mL^-1^) at 10°C.

**Fig 5:**
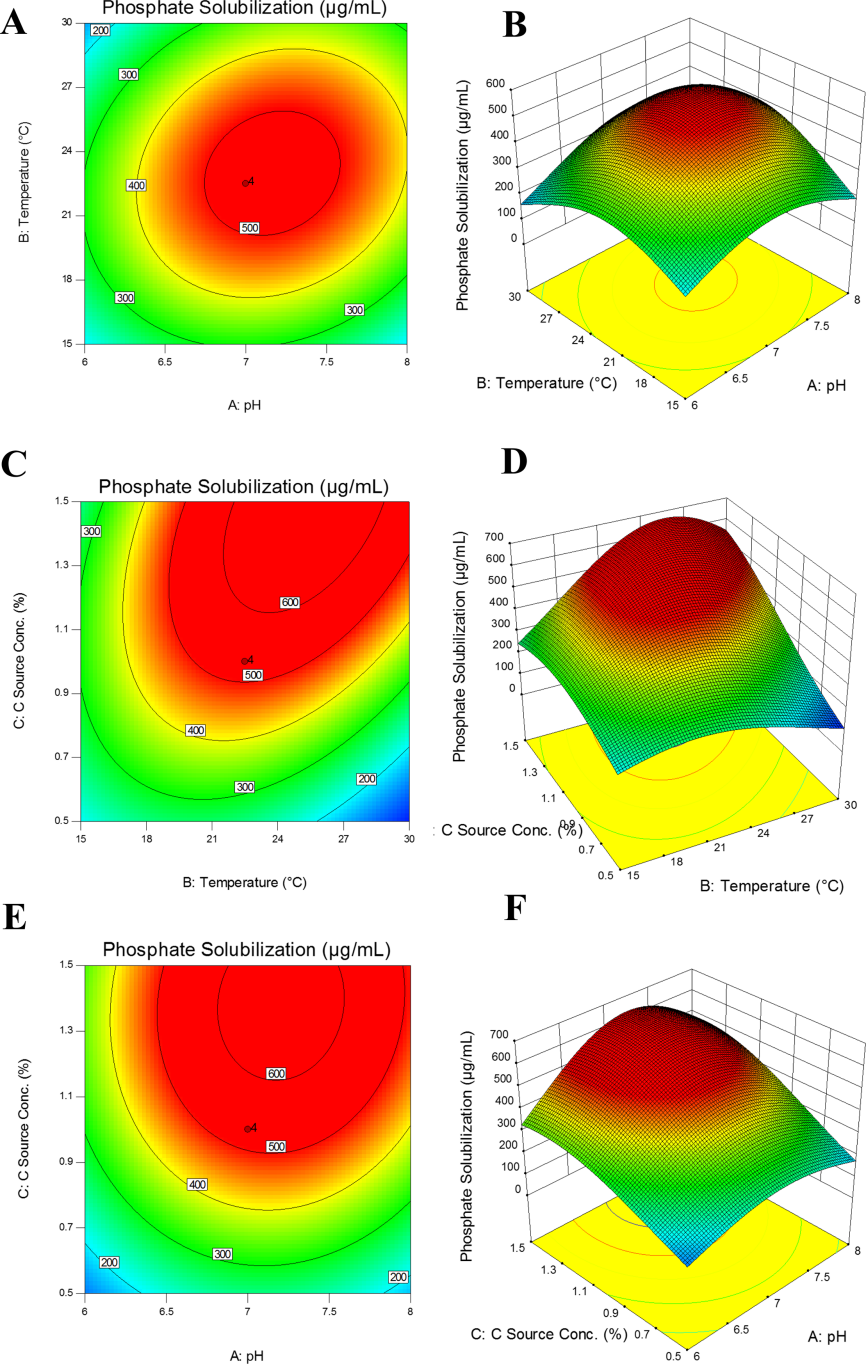
**Interaction among Temperature, pH and phosphate solubilizationusing Response surface (B,D,F) and contour (A,C,E) plotsat constant inoculums size** (~ 5 x 10^9^ CFU mL^-1^) Contour plot (a) and Response surface plot (b) depicting effect of mutual interaction of temperature and pH on Phosphate solubilization (%) at constant inoculum size (~ 5 x 10^9^ CFU mL^-1^) and 5 mg L^-1^ initial concentration of tri-calcium phosphate. There were three replicates for each treatment. A three factor/five level central composite design (CCD) full factorial with 18 runs was employed by using Design Expert software (trial version 10, Stat-Ease, Inc., MN, USA).

P solubilization increased at pH value initially set at 7.0. 3D Response surface graph and contour plot showed the interaction between pH, temperature and carbon source concentration and their combined effect on P solubilization was constructed through Design Expert 10.0 software (Fig 5). ANOVA from Design Expert software indicated that the model is significant (P<0.05) (S3 Table).

### Amplification of P solubilizing genes

**Fig 6:**
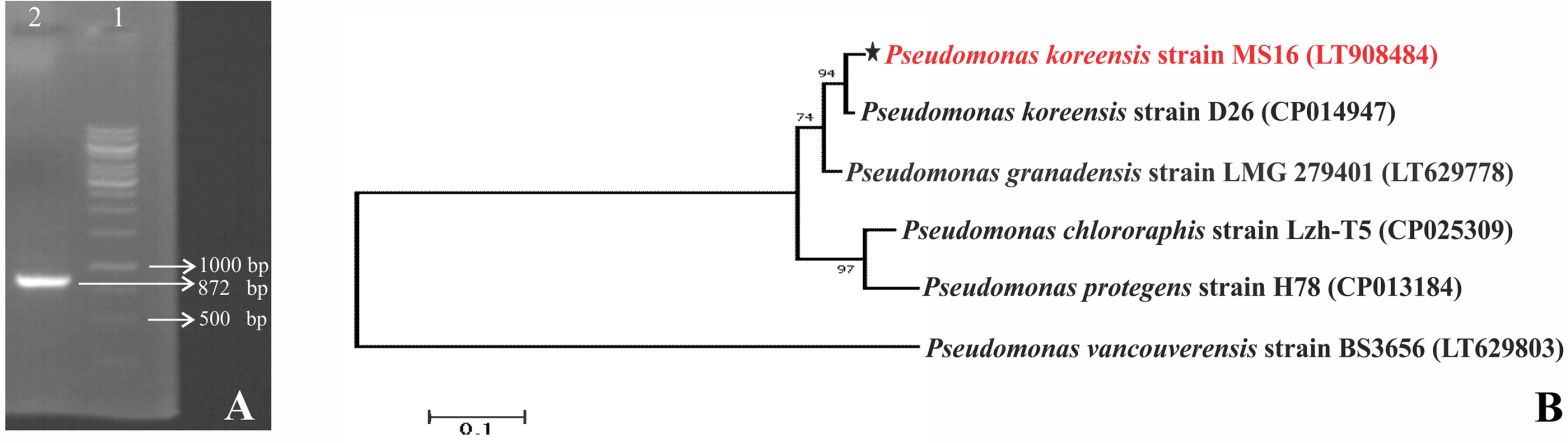
Amplification of *gcd* and sequence-based phylogenetic tree of PSB strains isolated from wheat rhizosphere. Amplification of 875 bp glucose dehydrogenase gene (gcd) using conserved PCR primers was observed. 1 Kb DNA ladder was used as marker (M). Bands were separated on 1% w/v agarose gel. The phylogenetic relationship of gcd gene sequence from *Pseudomonas* sp. MS16 was constructed by maximum likelihood method with help of MEGA6 software tool.

Glucose dehydrogenase gene (*gcd*) was PCR amplified (875 bp) from *Pseudomonas* sp. MS16 using conserved PCR primers (Fig 6). *Gcd* gene was not amplified from *Enterobacter* sp. MS32. Sequence analysis of *gcd* from *Pseudomonas* sp. MS16 showed maximum (83%) sequence homology with *gcd* of *Pseudomonas flourescens* (Accession no. KM251437) (Fig 6).Amplification of *pqqD* and *pqqE* genes using degenerate primers was not successful in both the tested strains.

### Bioassays for the detection of plant growth promoting activities in bacterial isolates

*Pseudomonas* sp. MS16 and *Enterobacter* sp. MS32 produced IAA as indicated by pink coloration upon reaction with Salkowski’s reagent using qualitative “spot test” (S2 Fig). When quantified with HPLC through ethyl acetate extraction method in tryptophan supplemented LB broth medium, higher IAA production (28.1 μg mL^-1^) was observed in *Enterobacter* sp. MS32 followed by *Pseudomonas* sp. MS16 (25.6 μg mL^-1^). Gibberellic acid (2.5–11.8 μg mL^-1^) was also produced in both of the tested strains (Table 2).

Nitrogenase activity was observed by color change of NFM medium from green to blue accompanied with pellicle formation in inoculated vials. Gas Chromatography further confirmed nitrogen-fixing activity in these isolates. *Enterobacter* sp. MS32 showed higher nitrogenase activity (19 nmol ethylene h^-1^ mg protein^-1^) followed by *Pseudomonas* sp. MS16 (8 nmol ethylene h^-1^ mg protein^-1^). Zinc solubilizing activity was observed in both the inoculated strains as indicated by the halo zone formation around colonies on tris minimal salt medium (S2 Fig). These strains were able to solubilize salt of Zinc i.e. Zinc Oxide. Solubilization index ranged between 2.8–3.3 mm. ACC deaminase activity was indicated by the growth of strains in vials supplemented with 0.5M ACC (S2 Fig and Table 2).

### Biosafety test for selection of PSB

Blood Agar test was conducted to screen bacteria for their biosafety assessment. Blood lysis or halo zone formation on blood agar medium was not observed in any of the selected PSB strains compared with control indicating that inoculated bacteria may not be pathogenic for humans and therefore, were safe to use for further studies.

### Effect of inoculated P solubilizing bacteria on wheat growth

**Fig 7:**
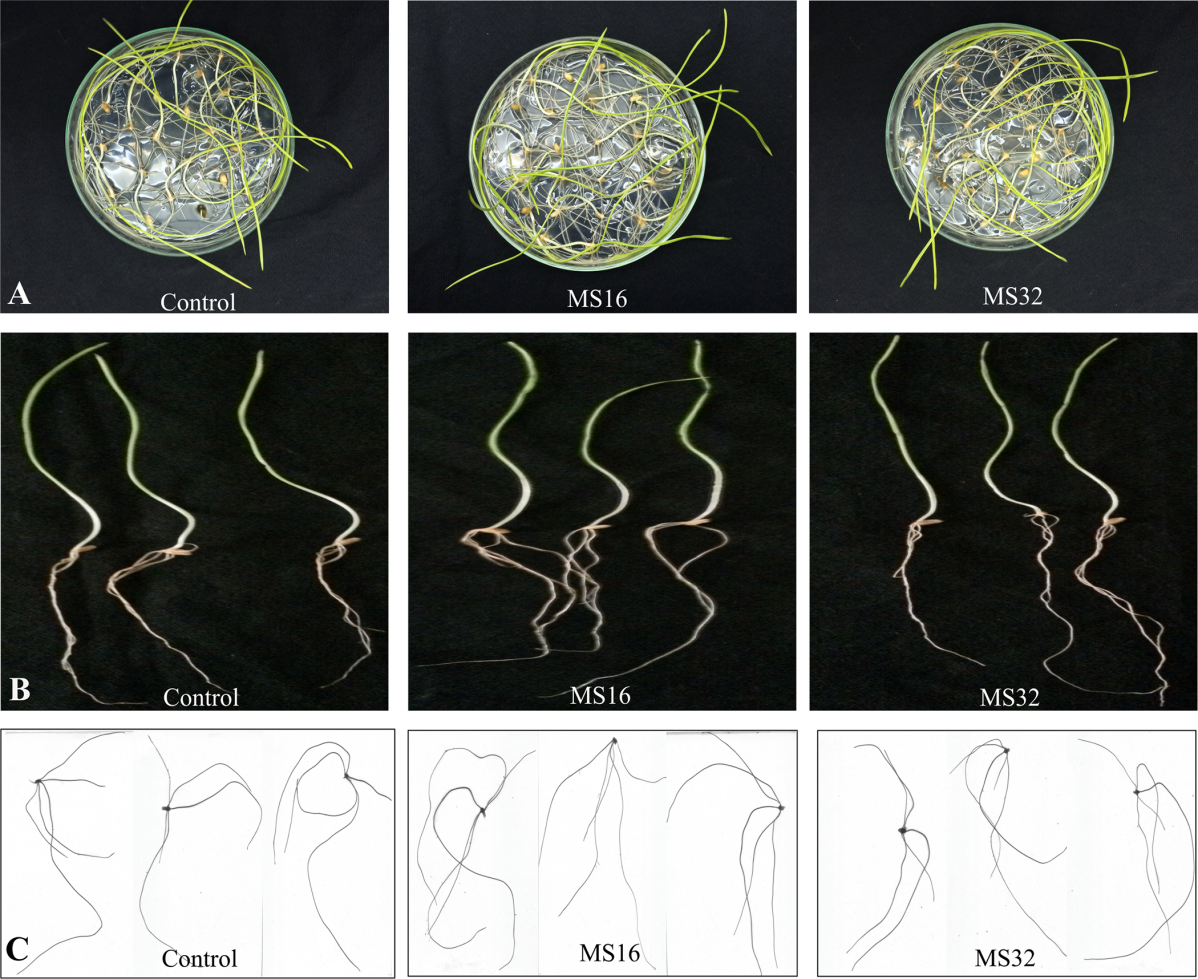
**Plate assay to study the effect of P solubilizing bacteria on wheat seedlings (A,B) and root growth (C)** Seeds were inoculated with PSB in water agar medium (0.3%) in a plate germination assay. Germination assay was carried out in a growth room and the data was recorded at 10 DPI. There were 21 seeds per plate. Seeds without inoculation were used as control. Plants were selected in triplicates for studying different root parameters through Rhizoscanner (EPSON Perfection V700 Photo, Epson America, Inc. USA), equipped with WinRHIZO software offered by Regent Instruments Co Canada.

A plate assay was carried out to study the effect of P solubilizing bacteria at early seedling stage of wheat variety ‘NN-Gandum-1’. Both *Pseudomonas* sp. MS16 and *Enterobacter* sp. MS32 had a positive effect on growth of wheat seedlings (Fig 7). Seedling Vigor index (VI) was calculated based on percent germination and hypocotyl/radical length (Table 3). Maximum VI (2760) was observed in seedlings inoculated with *Pseudomonas* sp. MS16 followed by *Enterobacter* sp. MS32 (VI: 2085).

**Table 3:** Root morphology data from Rhizoscanning.

Treatments	Root length (cm)	Root surface area (cm^2^)	Root diameter (mm)	Root volume (cm^3^)	Number of root tips	Number of forks	Area of projection (cm^2^)	No. of Crossings	Germination (%)	Vigor index
***Pseudomonas* sp. MS16**	52.41±6.69 **a**	7.50±1.45 **a**	0.45±0.03 **a**	0.08±0.02 **a**	58.33±8.02 **a**	58.50±1.10 **a**	2.39±0.46 **a**	4.67±1.15 **a**	95.2±4.7 **a**	2760±99 **a**
***Enterobacter* sp. MS32**	34.88±3.95 **b**	5.44±0.51 **b**	0.50±0.01 **a**	0.07±0.01 **a**	26.50±4.94 **b**	26.50±0.71 **b**	1.73±0.16 **b**	1.67±1.19 **b**	80.7±3.1 **b**	2085±64 **b**
**Un-inoculated Control**	42.80±3.36 **ab**	6.19±0.33 **ab**	0.46±0.05 **a**	0.06±0.02 **a**	24.50±7.71 **b**	21.00±2.41 **c**	1.97±0.10 **ab**	2.67±0.57 **b**	71.4±3.5 **c**	1233±78 **c**

Effect of PSB inoculation on various root morphology parameters. Seeds were inoculated with PSB in water agar medium in a plate assay and incubated at room temperature for 7 days. All values are an average of three biological replicates, ± represents standard deviation. Means followed by the same letter differ non-significantly at p = 0.05 according to LSD.

Rhizoscanning of wheat seedlings was carried out to observe the effect of P solubilizing bacteria on different parameters of root length and root morphology. Root length and surface area of roots were found to be increased after inoculation with P solubilizing bacteria. Various other root parameters i.e. root tips (26.5–58), and root surface area (5.4–7.5 cm^2^) was observed in roots of seedlings inoculated with *Enterobacter* sp. MS32 and *Pseudomonas* sp. MS16, respectively as compared to non-inoculated control (Fig 7 and Table 3).

### Evaluation of PSB for wheat yield parameters

Phosphate solubilizing bacteria improved wheat growth parameters in a pot experiment at NIBGE net house, Faisalabad. Inoculation of wheat variety NN-Gandum-1 with MS32 and MS16 showed increase in grain yield (2.9–3.2 g plant^-1^) and plant biomass (5.7–6.8 g plant^-1^), respectively as compared to 80% non-inoculated control. Higher seed P was observed in plants inoculated with MS32 (3.1%) and MS16 (3.2%) as compared to 80% non-inoculated control (2.7%) (Table 4).

**Table 4:** Effect of phosphate solubilizing bacteria on wheat growth in a pot experiment under net house conditions.

Treatment	^1^35 DAS				^2^120 DAS				
	Plant weight (g plant^-1^)	Shoot Length (cm)	Root Length (cm)	Viable (CFU g^-1^ soil)	Tillers (Tillers plant^-1^)	Shoot Length (cm)	Plant Biomass (g plant^-1^)	Grain Yield (g plant^-1^)	Plant Seed P content (%)
*Pseudomonas* sp. MS16	1.37±0.06 **a**	36.10±0.30 **a**	10.9±0.32 **a**	7.66 x10^7^	8.20±0.45 **a**	78.80±4.32 **a**	6.82±0.35 **a**	3.20±0.06 **a**	3.24±0.12 **a**
*Enterobacter* sp. MS32	0.87±0.03 **b**	29.00±0.36 **b**	6.97±0.15 **b**	1.46 x 10^7^	5.60±0.55 **b**	73.20±4.82 **a**	5.72±0.50 **b**	2.92±0.10 **b**	3.15±0.05 **a**
Control 80%	0.70±0.02 **c**	25.63±0.45 **c**	6.80±0.43 **b**	1.38 x 10^7^	4.40±0.55 **c**	79.00±1.58 **a**	4.58±0.48 **c**	2.31±0.08 **d**	2.77±0.12 **b**
Control 100%	0.60±0.02 **d**	24.87±0.25 **d**	5.87±0.15 **c**	3.32 x 10^7^	5.60±0.55 **b**	73.00±5.70 **a**	5.16±0.39 **bc**	2.59±0.19 **c**	2.79±0.14 **b**

^1, 2^Effect of bacterial inoculation on various wheat growth parameters at 35 days after sowing (DAS) and at 120 DAS in a pot experiment.

Control 80% and Control 100% represents non-inoculated controls supplemented with 80% of the recommended dose of P fertilizer and full recommended dose of P fertilizer, respectively. *Pseudomonas* sp. MS16 and *Enterobacter* sp. MS32 (1 x10^9^ CFU mL^-1^) were seed-inoculated before sowing. Inoculated pots were supplemented with 80% of recommended dose of DAP fertilizer.Means are the average of six replicates arranged in CRD. Means followed by the same letter differ non-significantly at p = 0.05 according to LSD.±represents the standard deviations (SD). Plants were grown in earthen pots (Diameter: 12 inch, Height: 14 inch) containing 12 Kg soil. Three plants were grown per pot.

Based on the higher growth promoting effects on wheat in pot experiment, *Pseudomonas* sp. MS16 was selected for further evaluation in microplots for two successive years (wheat growing season 2016–17 and 2017–18). First year data revealed significant improvement in grain yield (0.42 Kg plot^-1^), plant biomass (1.38 Kg plot^-1^) and number of tillers (~14 tillers plant^-1^) of the inoculated plants as compared with non-inoculated 80% control (Table 5). Second year (2017–18) data also showed a pronounced effect of *Pseudomonas* sp. MS16 on grain yield (0.53 Kg plot^-1^) and plant biomass (1.36 Kg plot^-1^) as compared to non-inoculated 80% control (Table 5). Higher seed P (3.23–3.49%) was observed in inoculated plants as compared to 80% non-inoculated control during first and second wheat growing seasons, respectively (Table 5).

**Table 5:** Effect of inoculated phosphate bacteria on plant growth and yield of wheat in microplots.

	Treatment	Number of Tillers (Tillers plant^-1^)	Plant Height (cm)	Root length (cm)	Plant Biomass (Kg plot^-1^)	Grain Yield (Kg plot^-1^)	Plant Seed P content (%)
***Winter Season 2016–17***	*Pseudomonas* sp. MS16	14.6±1.2 **a**	99.73±2.69 **a**	13.95±1.41 **a**	1.38±0.14 **a**	0.42±0.09 **a**	3.49±0.12 **a**
	Control 80%	10.7±0.8 **b**	98.40±2.55 **a**	10.38±1.25 **ab**	1.27±0.01 **b**	0.35±0.01 **b**	3.04±0.15 **b**
	Control 100%	11.0±1.0 **b**	99.00±2.65 **a**	11.65±0.92 **b**	1.30±0.03 **ab**	0.40±0.07 **a**	3.11±0.11 **b**
***Winter Season 2017–18***	*Pseudomonas* sp. MS16	13.3±0.52 **a**	98.49±0.81 **a**	13.96±0.55 **a**	1.36±0.12 **a**	0.53±0.03 **a**	3.23±0.05 **a**
	Control 80%	10.3±0.57 **c**	97.90±0.95 **a**	12.40±1.11 **a**	1.01±0.11 **b**	0.42±0.04 **b**	3.11±0.04 **b**
	Control 100%	11.6±0.58 **b**	97.39±0.87 **a**	9.40±1.21 **b**	1.11±0.10 **ab**	0.43±0.02 **b**	3.05±0.07 **c**

Wheat plants were grown in microplots during crop season 2016–17 under net house conditions. Control 80% and Control 100% represents non-inoculated controls supplemented with 80% of the recommended dose of P fertilizer and recommended dose of P fertilizer, respectively. *Pseudomonas* sp. MS16 (1 x10^9^ CFU mL^-1^) was seed-inoculated before sowing. Inoculated plots were supplemented with 80% of recommended dose of DAP fertilizer. Means are an average of three replicates arranged in RCBD. Means followed by the same letter differ non-significantly at p = 0.05 according to LSD. ± represents the standard deviations (SD). Each microplot has an area of 2.25 m^2^ and 54 plants plot^**-**1^ were grown.

Field trials were conducted at two different locations to assess the effect of PSB MS16 on growth of two wheat varieties NN-Gandum-1 and Fakhr-e-Sarhad under different environmental conditions of Faisalabad and Peshawar, respectively. Data analysis showed an increase in number of tillers (436–530 tillers m^-2^), plant biomass (14505–14280 Kg hac^-1^) and grain yield (5453 Kg hac^-1^–5646 Kg hac^-1^) over non-inoculated 80% control at NIBGE, Faisalabad and NIFA, Peshawar, respectively (Table 6).

**Table 6:** Effect of inoculated bacteria on various plant growth and yield parameters of wheat in field trials.

Sites	Treatments	No. of tillers (tillers m ^-2^)	Shoot Length (cm)	Root length (cm)	Plant biomass (Kg ha^-1^)	Grain yield (Kg ha^-1^)
**Peshawar, KPK province**	Inoculated	436±4.93 **a**	106.35±4.34 **a**	10.3±0.35 **a**	14280±1137.1 **a**	5646±61.11 **a**
	80% Control	352±11.6 **c**	106.65±4.17 **a**	9.65±0.21 **b**	11035±664.80 **b**	4813±56.86 **b**
	100% Control	373±4.51 **b**	106.30±2.31 **a**	8.75±0.75 **b**	14035±718.95 **a**	4970±160.9 **b**
**Faisalabad, Punjab province**	Inoculated	530±7.09 **a**	108.25±3.63 **a**	13.9±0.35 **a**	14505±642.98 **a**	5453±92.91 **a**
	80% Control	461±7.29 **b**	109.30±1.62 **a**	10.1±0.57 **b**	11650±479.58 **b**	4590±185.2 **b**
	100% Control	481±11.1 **b**	107.25±2.33 **a**	11.8±0.42 **b**	13866±315.54 **a**	4946±126.6 **b**

Effect of PSB on Wheat growth parameters at Peshawar and Faisalabad was studied during winter season 2017. Control 80% and Control 100% represents non-inoculated controls supplemented with 80% of the recommended dose of P fertilizer and recommended dose of P fertilizer, respectively.*Pseudomonas* sp. MS16 (1 x10^9^ CFU mL^-1^) was seed-inoculated before sowing. Inoculated plots were supplemented with 80% of recommended dose of DAP fertilizer. Data is an average of five replicates. Data was taken at 120 DAS.±represents Standard Deviation. Means are an average of five replicates arranged in RCBD.Means with significant difference (P<0.05) among treatments is represented by different letter.

### Effect of PSB on P uptake of wheat and soil nutrients

#### Estimation of phosphorus in plant samples

Seed P contents (2.2–3.8%) of field-grown wheat plants inoculated with MS16 was significantly (p<0.05) higher as compared to non-inoculated 80% control (1.99–3.34%) at NIFA, Peshawar and NIBGE, Faisalabad, respectively (Table 7).

**Table 7:** Effect of PSB inoculation on P uptake ofwheat and soil nutrients in field trials.

Site	Treatment	Plant Seed P content (%)	Soil available P (μg g^-1^ soil)	Soil Phosphatase Activity (μmoles g^-1^ soil hr^-1^)	Soil Potassium Content (μg g^-1^ soil)	Soil Sodium content (μg g^-1^ soil)
**Peshawar,** **KPK province**	Inoculated	2.20±0.08 **a**	2.10±0.09 **a**	25.43±0.08 **a**	40.95±1.95 **a**	42.47±1.16 **a**
	Control 80%	1.99±0.11 **b**	1.56±0.13 **c**	21.55±0.11 **c**	34.45±0.81 **b**	30.27±0.59 **b**
	Control 100%	2.14±0.02 **ab**	1.84±0.16 **b**	22.02±0.02 **b**	33.54±1.56 **b**	31.67±0.47 **b**
**Faisalabad,** **Punjab province**	Inoculated	3.84±0.11 **a**	1.99±0.07 **a**	21.24±0.22 **a**	33.67±2.15 **ab**	39.64±0.58 **a**
	Control 80%	3.34±0.06 **b**	1.53±0.06 **b**	19.30±0.91 **b**	34.19±1.25 **a**	8.820±0.66 **c**
	Control 100%	3.68±0.06 **a**	1.66±0.08 **b**	20.25±0.80 **ab**	30.81±0.39 **c**	18.02±0.66 **b**

Effect of PSB on wheat P uptake and soil nutrients at Peshawar grown during winter season 2017. Data is an average of five replicates. Data was taken at 120 Days after sowing (DAS). ± represents Standard Deviation. Means with significant difference (P<0.05) among treatments is represented by different letter. *Pseudomonas* sp. MS16 (1 x10^9^ CFU mL^-1^) was seed-inoculated before sowing. Inoculated plots were supplemented with 80% of recommended dose of DAP fertilizer.

#### Estimation of phosphorus, sodium, potassium and phosphatase activity in soil samples

Phosphorus content of soil was measured by spectrophotometric analysis of digested samples. In pot experiment, increase in soil available P (2.5–2.7 μg g^-1^ soil) and phosphatase activity (21.3–22.5 μmoles g^-1^ soil h^-1^) was observed with PSB MS32 and MS16, respectively (Table 4 and S4 Table). Whereas in microplots, increase in soil available P (3.2–2.7 μg g^-1^ soil) and phosphatase activity (25.3–23.2 μmoles g^-1^ soil h^-1^) was observed in plants inoculated with MS16 as compared to 80% non-inoculated control during 2016–17 and 2017–18, respectively (Table 5 and S4 Table).

Soil available phosphorus content (1.9–2.1 μg g^-1^), potassium (40.9–33.6 μg g^-1^ soil) and sodium (42.4–39.6 μg g^-1^ soil) contents enhanced in soils inoculated with *Pseudomonas* sp. MS16 in field trials at NIBGE, Faisalabad and NIFA, Peshawar, respectively (Table 7).

Inoculation effects of PSB MS16 and MS32 on soil phosphatase was studied in pot experiment. While, the soil phosphatase activity of PSB MS16 was further assessed in microplots and field experiments. Light pink color formation was observed when phosphatase reacted with p-nitrophenyl. In spectrophotometric analysis, maximum phosphatase activity (21.2–25.4 μmoles g^-1^ soil hr^-1^) was observed in *Pseudomonas* sp. MS16 inoculated soils with 10% and 18% increase at Faisalabad and Peshawar, respectively (Table 7).

### Detection and colonization of inoculated PSB

**Fig 8:**
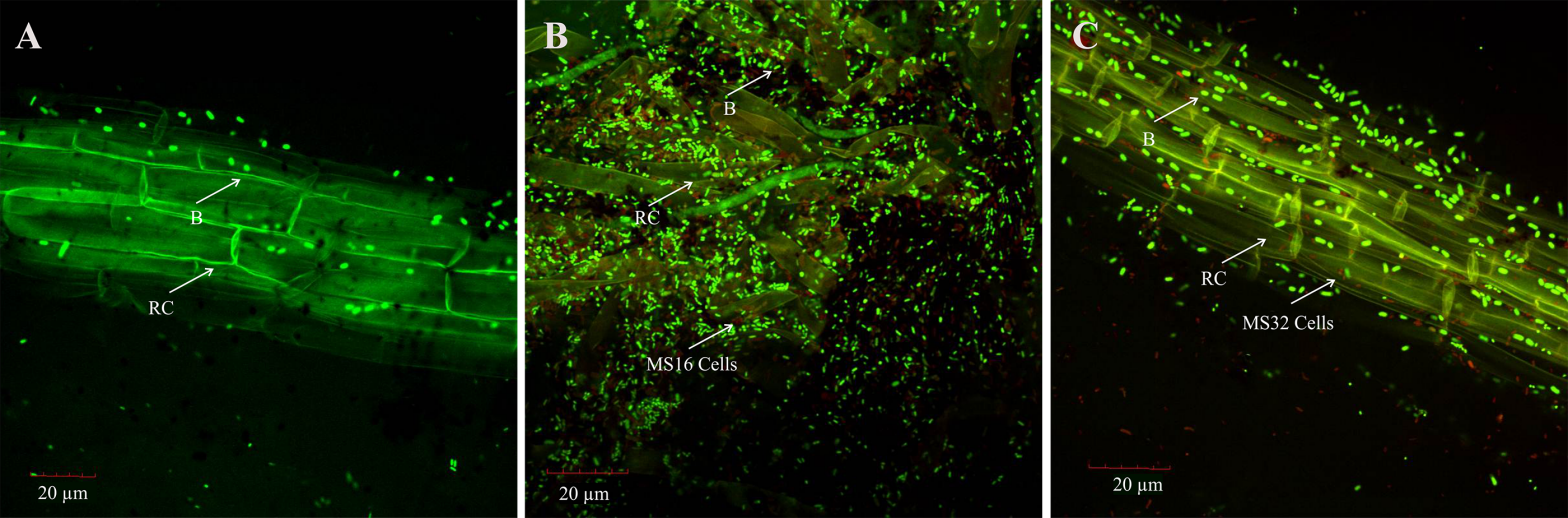
**CLSM images of root sections showing *in situ* detection of PSB** A: Control with native bacteria, B: Root colonization of *Pseudomonas* sp. MS16 red cells using Cy3-labelled probe PSM specific for *Pseudomonas* spp., C:, B: Root colonization of *Enterobacter* sp. MS32 using Cy3 labelled GAM42a. RC represents root cell and B represents native bacterial cells.

**Fig 9:**
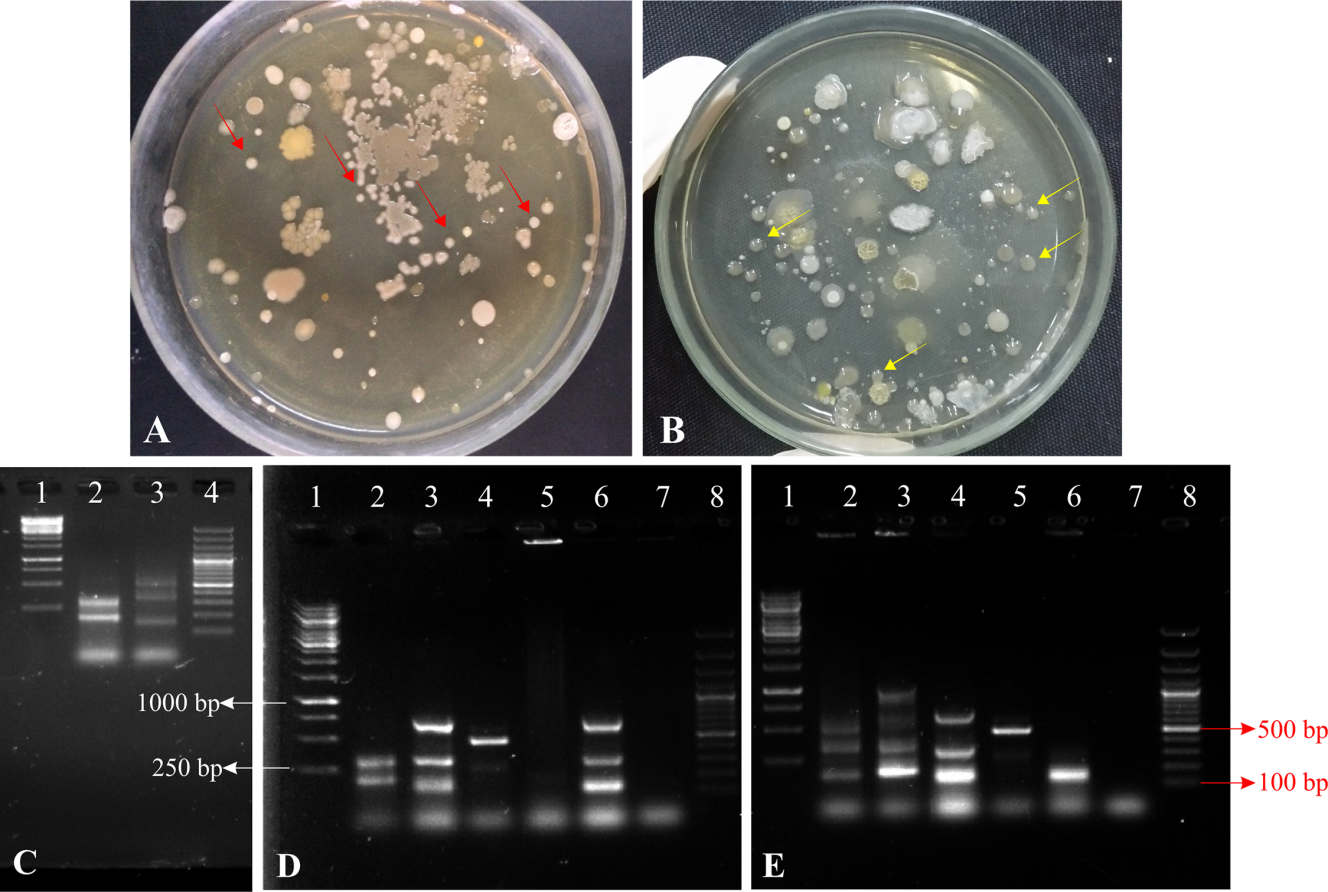
Detection of inoculated P solubilizing bacteria at 35 DAS. Viable count after 35 days to study the survival of *Pseudomonas* sp. MS16 (**A**) and *Enterobacter* sp. MS32 (**B**) on the roots of wheat plants. Yellow, circular and shiny colonies indicated by red arrows show the presence of *Pseudomonas* sp. MS16 while Off-white, circular, and shiny colonies indicated by yellow arrows show presence of *Enterobacter* sp. MS32 among total indigenous bacterial soil population. Amplification of colony of isolated bacteria by using BOX primer in PCR (**C**). BOX-PCR of re-isolated colonies obtained from rhizosphere of plants grown in pots inoculated with; (A) Pseudomonas sp. MS16; Lane 1: 1 Kb DNA Ladder, Lane 2: Re-isolated colony of Pseudomonas sp. MS16; Lane: 3-6 Colonies of other isolates obtained, Lane:7 Negative control, Lane 8: 100bp DNA Ladder. (B) Enterobacter sp. MS32; Lane 1: 1 Kb DNA Ladder, Lane 2: Re-isolated colony of Enterobacter sp. MS32, Lane: 3-6 Colonies of other isolates obtained, Lane:7 Negative control, Lane 8: 100bp DNA Ladder.

To confirm the presence of inoculated bacteria in the rhizosphere of wheat variety NN-Gandum-1 grown in pots under net house conditions, bacterial population was recorded at 35 days after sowing. Viable count, BOX-PCR and FISH confirmed the presence of inoculated bacteria (Fig 8 and Fig 9). The re-isolated colonies of *Pseudomonas* sp. MS16 and *Enterobacter* sp. MS32 were identified based on morphological characters along with other characteristics i.e. P solubilization (MS16: 280.2 μg mL^-1^ and MS32: 136.3 μg mL^-1^) and IAA production (MS16: 23.2 μg mL^-1^ and MS32: 26.3 μg mL^-1^) as compared with those of the pure cultures BOX-PCR pattern of the re-isolated colonies was found to be similar as that of pure culture. Digital image analysis of FISH-CLSM images obtained from *Pseudomonas* sp. MS16-inoculated roots showed total bacterial population up to ~1.52 x 10^1^ cells μm^-2^ and red colored cells of *Pseudomonas* spp. up to ~8.9 cells μm^-2^ (Fig 8B). Total bacterial population in *Enterobacter* sp. MS32 inoculated roots reached up to ~1.3 x 10^1^ cells μm^-2^ along with ~3.12 cells μm^-2^ red colored cells belonging to *gamma Proteobacteria* (Fig 8C).

**BOX-PCR of re-isolated colonies obtained from rhizosphere of plants grown in pots inoculated with; (A) *Pseudomonas* sp. MS16**; Lane 1: 1 Kb DNA Ladder, Lane 2: Re-isolated colony of *Pseudomonas* sp. MS16; Lane: 3–6 Colonies of other isolates obtained, Lane:7 Negative control, Lane 8: 100bp DNA Ladder. (**B) *Enterobacter* sp. MS32**; Lane 1: 1 Kb DNA Ladder, Lane 2: Re-isolated colony of *Enterobacter* sp. MS32, Lane: 3–6 Colonies of other isolates obtained, Lane:7 Negative control, Lane 8: 100bp DNA Ladder.

## Discussion

Due to limiting amount of rock phosphate and high production cost, use of microbes has emerged as an alternate strategy for supplying phosphorus to plants. Keeping in view the importance of low cost production of wheat with high yield using environmentally safe management approach, the present study was designed to isolate P solubilizing bacteria from wheat rhizosphere of different soils and find their optimal efficacy under different growth and environmental conditions. Additionally, the mechanisms and genes involved in P solubilization were explored. To get the ultimate goal of developing phosphatic bio-fertilizers using phosphobacteria, selected *Pseudomonas* sp. MS16 were evaluated in field experiments at Faisalabad, Punjab and Peshawar, KPK, Pakistan.

P solubilizing bacteria were isolated from wheat rhizospheric soil of Peshawar and southern Punjab region. Previous studies reported the occurrence of PSB in upper or central area of Punjab [70] while Peshawar, KPK and southern Punjab region were not explored earlier as no microbiome data of these wheat growing areas is available. The presence of P solubilizing bacterial population in these un-explored soils indicated the opportunity to use these native beneficial bacteria as inoculum for cost effective sustainable wheat production.

Different morphotypes isolated on nutrient agar, Pikovskaya agar and NBRIP agar media were screened *in vitro* to utilize tri-calcium phosphate (TCP) as an insoluble source. Fifteen isolates solubilized inorganic phosphate with solubilization index ranged from 2.4 to 5.8 in plate assay Only 9% of the tested isolates was found to have P solubilizing activity. Previous studies also reported very low frequency of P solubilizing bacteria in total bacterial population [71]. Comparative studies of *in vitro* plate assay and spectrophotometric quantification using Pikovskaya and NBRIP showed that more P solubilizing activity was observed on NBRIP as compared to Pikovskaya, therefore, NBRIP can be regarded as more efficient phosphate solubilization medium [72].

Among the isolated bacteria, two efficient P solubilizing bacteria MS16 and MS32 were selected for further studies. Light microscopy and Gram’s staining showed that these isolates were motile, rod shaped and Gram negative bacteria. 16S rRNA gene sequencing and phylogenetic analysis identified MS16 and MS32 as *Pseudomonas* sp. and *Enterobacter* sp., respectively. The identified bacterial genera have been reported as plant growth promoting bacteria [8, 73–76].

Both selected PSB solubilized sufficient P (136–280 μg mL^-1^) in quantitative assay with significant pH decrease up to 3.58 from 7 in liquid medium. Phosphate solubilizing *Pseudomonas* sp. strain MS16 did not show high solubilization index (SI) on both TCP added media (Pikovskaya and NBRIP). However, it showed significant phosphate solubilization in liquid medium indicating that visible halo zone formation cannot serve as a reliable criterion for isolating PSB as many isolates that didn’t form halo zone on agar medium, solubilized insoluble phosphates in liquid culture medium [72]. Similar results were also reported by Gupta et al. [33].

Organic acid production is the principal mechanism adopted by PSB for inorganic phosphate solubilization. Most Gram negative bacteria show glucose dehydrogenase activity that can solubilize phosphate by extracellular oxidation of glucose to gluconic acid [77]. In the present study, both the tested PSB produced acetic, citric, gluconic, malic, oxalic and succinic acid to a variable extent in culture supernatant within 7 days as detected by HPLC. Potential PSB *Pseudomonas* sp. MS16 showed the significant production of gluconic acid which may be one of its main P solubilizing mechanism as organic acid production is directly correlated with P solubilizing efficiency of microbes [70].

*In vitro* biological assays revealed that both the selected PSB produced IAA showing their potential to enhance plant growth by using growth regulators. IAA production was reported to improve root growth parameters i.e. surface area of roots and length and thus soil nutrients and water uptake efficiency is improved [78]. Acetylene reduction assay detected *Pseudomonas* sp. MS16 as nitrogen fixing bacteria that may contribute to enhance N content of wheat. Previous studies indicated large population of wheat associated nitrogen fixers in the soils [71]. Zinc is one of the key nutrient required which is required in a small concentration for proper growth and regulation of important metabolic reactions in a plant. Phosphate solubilizing microorganisms can help solubilize fixed zinc by productions of protons and chelating compounds. Among reported zinc solubilizers, *Pseudomonas* sp. and *Bacillus* sp. are most abundant [79–81]. *Pseudomonas* sp. MS16 and *Enterobacter* sp. MS32 with Zn solubilizing ability (SI: 2.8–3.3) may have potential to solubilize multi nutrients under stress or nutrient deficient soil conditions. *Pseudomonas* sp. MS16 and *Enterobacter* sp. MS32 with ACC deaminase activity suggest that they can help plants in lowering down many stresses that can have a detrimental effect on plant health and growth. Studies have reported that the use of ACC deaminase producing bacteria may help to lower the intensity of stress [82].

The effect of carbon source or sugars used on extent of P solubilizing efficiency was evaluated for the potential PSB strains as the level of P solubilization by PSB varies with the carbon source used in medium [83]. Glucose was found to be optimal carbon source supporting higher P solubilization *in vitro*.

*Pseudomonas* sp. MS16 solubilized more P using glucose as compared to *Enterobacter* sp. MS32; therefore, interaction of pH, temperature and carbon (glucose) source concentration and their combined effect on P solubilization by MS16 was determined using RSM. RSM plot showed that phosphate solubilization increased with incubation temperature (22.5 ^o^C) and pH (7) up to an optimum point where further increase in temperature or pH may result in decrease of P solubilization activity. Carbon source concentration also had a positive correlation with phosphate solubilization.

Degenerate primers pqqF/ pqqR [16] were used for the amplification of the pqqE gene in PSB MS16 and MS32 using described conditions [70] in repeated PCR experiments but we could not get any amplification. This showed that although insoluble P is being solubilized by these strains but the genes might be different from those reported earlier and some other primers targeting different portion of the gene may be used to amplify the pqq gene. P solubilization takes place by direct oxidation of glucose into gluconic acid by the membrane bound glucose dehydrogenase (*gcd*) [84]. Amplification and sequencing of *gcd* gene of PSB *Pseudomonas* sp. MS16 suggested that this gene might contribute for its P solubilization activity. To date, this is the first molecular study identifying the presence of gcd gene in wheat rhizosphere associated phosphate solubilizing bacteria. Earlier studies have focused on molecular studies on PSB from environmental samples and different crops [16, 21, 66, 85]

*Pseudomonas* sp. MS16 and *Enterobacter* sp. MS32 was evaluated *in vitro* in a plate assay and *in vivo* under field conditions. *In vitro* plate assay showed a positive effect of inoculation on germination and seedling growth of wheat. *Pseudomonas* sp. MS16 and *Enterobacter* sp. MS32 improved different root parameters i.e. root volume (6–25%) and root tips (8–138%) of inoculated seedlings as compared to non-inoculated control while root surface area was found to be increased (22%) by only *Pseudomonas* sp. MS16 (Fig 7)”.

In pot experiments, bacterial inoculation of wheat with MS32 and MS16 showed potential to enhance plant growth promotion as indicated by increased grain yield (26.4–38.5%) and plant biomass (24.8–48.9%), respectively, as compared to 80% non-inoculated control (Table 4). Higher growth improvement and plant P (16.9% increase) was observed in plants inoculated with *gcd* containing *Pseudomonas* sp. MS16 as compared to 80% non-inoculated control. Afshan et al. also reported wheat growth promotion with PGPR inoculation [71].

Total bacterial population in soil of inoculated pots as compared to that of un-inoculated control pots showed no negative effect of inoculant on natural bacterial population. Absence of non-hemolytic activity of PSB on blood agar indicated that these strains are not pathogenic [48] but further biosafety studies will be carried out for their safe application.

Based on the higher growth promoting effects on wheat in pot experiment, MS16 was selected for further evaluation in microplots and field (Table 5). Evaluation of MS16 in microplots for two successive years (wheat growing season 2016–17 and 2017–18) showed significant increase in grain yield (20–26%) and plant P (14.8–3.85%) of wheat variety NN-Gandum-1 as compared to 80% non-inoculated control. The grain yield (5–23%) and plant P (12–5.9%) was found to be increased as compared to 100% non-inoculated control supplemented with recommended doses of N and P in two consecutive years, respectively. The enhanced growth promotion with plant P of wheat variety NN-Gandum-1 is an indicator of bio-efficacy of *Pseudomonas* sp. MS16 (Table 5).

The present study has demonstrated the likely contribution of inoculated P solubilizing *Pseudomonas* sp. MS16 towards growth promotion of wheat under field conditions at NIFA, Peshawar and NIBGE, Faisalabad. Among investigated yield characters, grain yield (13.6–17%) and plant P content (2.6–14.9%) of wheat variety Fakhr-e-Sarhad grown at NIFA, Peshawar were found to be increased with *Pseudomonas* sp. MS16 inoculation as compared to 100% and 80% non-inoculated controls, respectively. Whereas, grain yield (10.2–18%) and plant P content (4.3–14.9%) of wheat variety NN-Gandum-1 grown at NIBGE, Faisalabad were found to be increased with *Pseudomonas* sp. MS16 inoculation as compared to 100% and 80% non-inoculated controls, respectively (Tables 6, 7). The significant increase in wheat plant growth and P contents by inoculated PSB is a clear indicative of the fact that the bacterial isolates have been able to provide better nutrient uptake [51, 53, 71]

The inoculated PSB strain MS16 caused an increase in available P content along with other soil nutrients i.e. exchangeable K and Na content. Soil fertility increases with enhanced soil nutrients contents. As reported earlier [86, 87], when rhizospheric microorganisms were introduced to potato, different leguminous and non-leguminous plants, they gradually increased the crop production and soil fertility over the years [88, 89].

Phosphatase activity in soil after harvesting the PSB-inoculated wheat plants was found to be higher as compared to that of the soil of non-inoculated control plots. Earlier studies have reported that enzyme activities especially soil phosphatase activity can be compared with soil biological activities including microbial biomass [90, 91]. Present study indicated that the inoculation of PSB might have a positive effect on native microbial flora of soils. In addition, phosphatase enzyme releases P from inorganic sources leading to higher P solubilization and higher P uptake by plants [92].

Root colonization by PSB was observed with BOX, FISH, CLSM as well as by conventional viable count method. Morphological characteristics of the inoculated bacteria also facilitated detection from the roots showing colonization and rhizosphere competence [49].

To the best of our knowledge, this is the first report focusing on *gcd* containing phosphate solubilizer from wheat rhizosphere. This study has investigated the effect of phosphate solubilizing bacteria isolated from un-explored soils and their effect on wheat growth and P uptake. Phosphate solubilization activity of the most efficient PSB *Pseudomonas* sp. MS16 was optimized for different environmental conditions for large-scale inoculum production. This strain with plant growth promoting attributes utilized different sugars and showed significant production of gluconic acid. This bacterium also increased the wheat yield as compared to non-inoculated controls along with P uptake and P release in soil. Viable, BOX-PCR and FISH indicated viability and rhizosphere competence of PSB in soil. Therefore, gluconic acid producing *Pseudomonas* sp. MS16 may be considered as promising candidate for the production of phosphorus solubilizing bio-fertilizer for wheat under P deficient soils.

## Supporting information

S1 Fig Solubilization index of PSB on Pikovaskaya and NBRIP medium. Solubilzation index of PSB was measured on two different media. Data is an average of three replicates. Error bars represent ±S.D. Means with significant difference (P<0.01) among treatments is represented by different letter.
(TIF)

S2 Fig Plant Growth promoting traits of PSB. PGP traits of PSB was detected by IAA production as indicated by pink coloration upon reaction with Salkowski's reagent (A), Solubilization of zinc salts using tris minimal salt medium containing insoluble salts of zinc (B), and detection of ACC deaminase activity in vials containing 30 μL of 0.5 M ACC as a sole nitrogen source in 5 mL DF salt minimal medium (C). +C represent positive control while–C represent negative control (without inoculation).
(TIF)

S3 Fig Effect of inoculated PSB on wheat growth compared with non-inoculated control at 35 DAS in pot experiment. A: Root/ Shoot length at 35 days after sowing B: Wheat growth in pots inoculated with compared PSB as compared to un-inoculated controls.
(TIF)

S1 Table Physico-chemical properties of soils collected from experimental field sites prior to sowing. All information presented in this table are from current study. Soil was collected from experimental sites and analyzed for different physico-chemical properties. All values are an average of three biological replicates.
(DOCX)

S2 Table Identification of PSB based on 16S rRNA gene sequence analysis database. Sequences of Strains were analyzed using NCBI GenBank database.
(DOCX)

S3 Table Analysis of variance for phosphate solubilization response (μg mL^-1^) using response surface methodology. R2 = 0.9159 Adjusted R2 = 0.8212
Predicted R2 = 0.3584
Adequate precision = 7.909
* Significant at p<0.05.
(DOCX)

S4 Table Effect of PSB inoculation on soil nutrients in pots and microplots. Effect of PSB on soil nutrients in pots and microplots. Data is an average of three replicates. Data was taken at 120 Days after sowing (DAS). ± represents Standard Deviation. Means with significant difference (P<0.05) among treatments is represented by different letter.
(DOCX)
