# Supplementary figures and images for: Phosphate solubilizing bacteria with glucose dehydrogenase gene for phosphorus uptake and beneficial effects on wheat
Source: PLoS One. 2018 Sep 21;13(9):e0204408. doi: 10.1371/journal.pone.0204408 (PMC6150522; doi:10.1371/journal.pone.0204408)

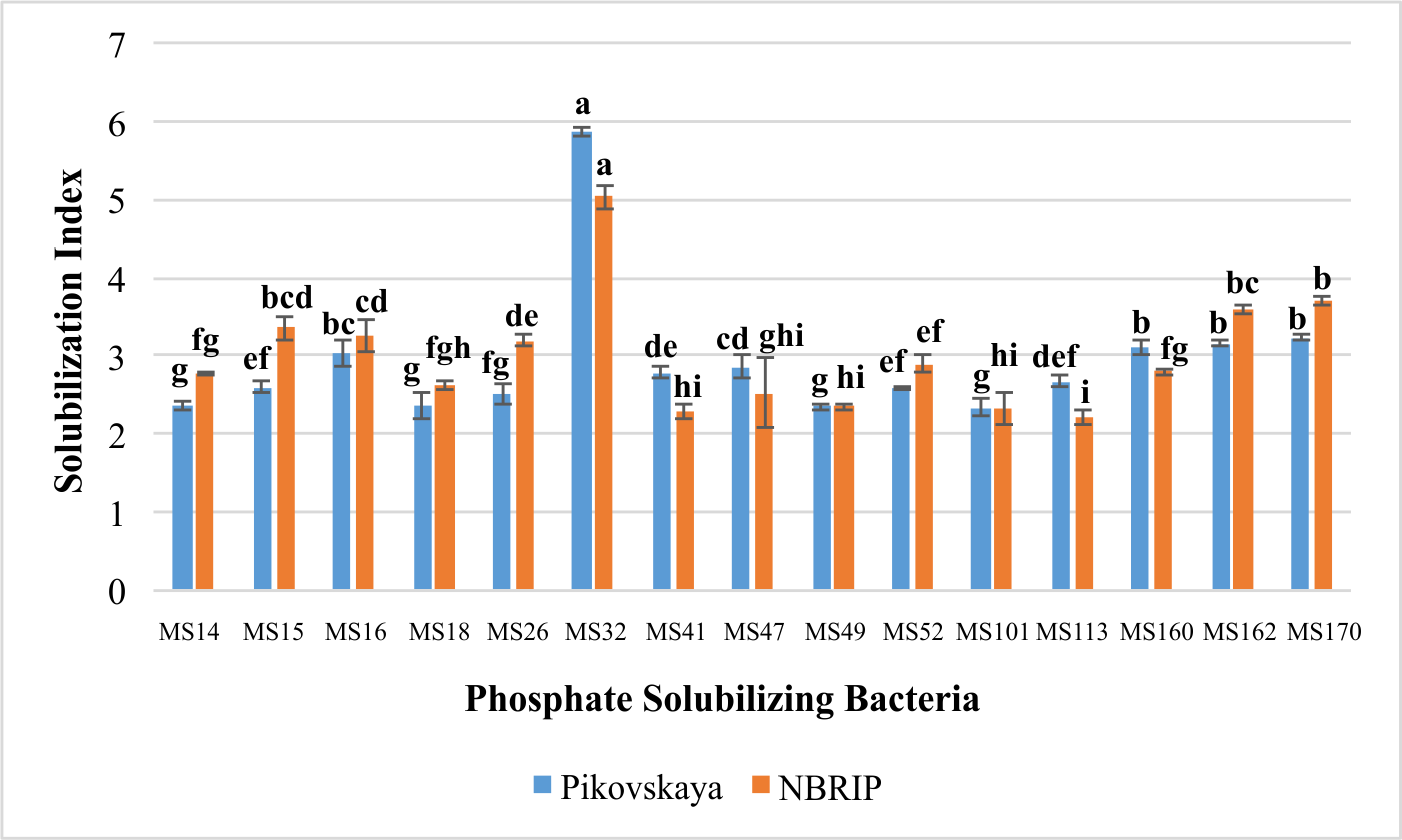

Supplement: S1 Fig — Solubilzation index of PSB was measured on two different media. Data is an average of three replicates. Error bars represent ±S.D. Means with significant difference (P<0.01) among treatments is represented by different letter. (TIF) [file pone.0204408.s001.tif]

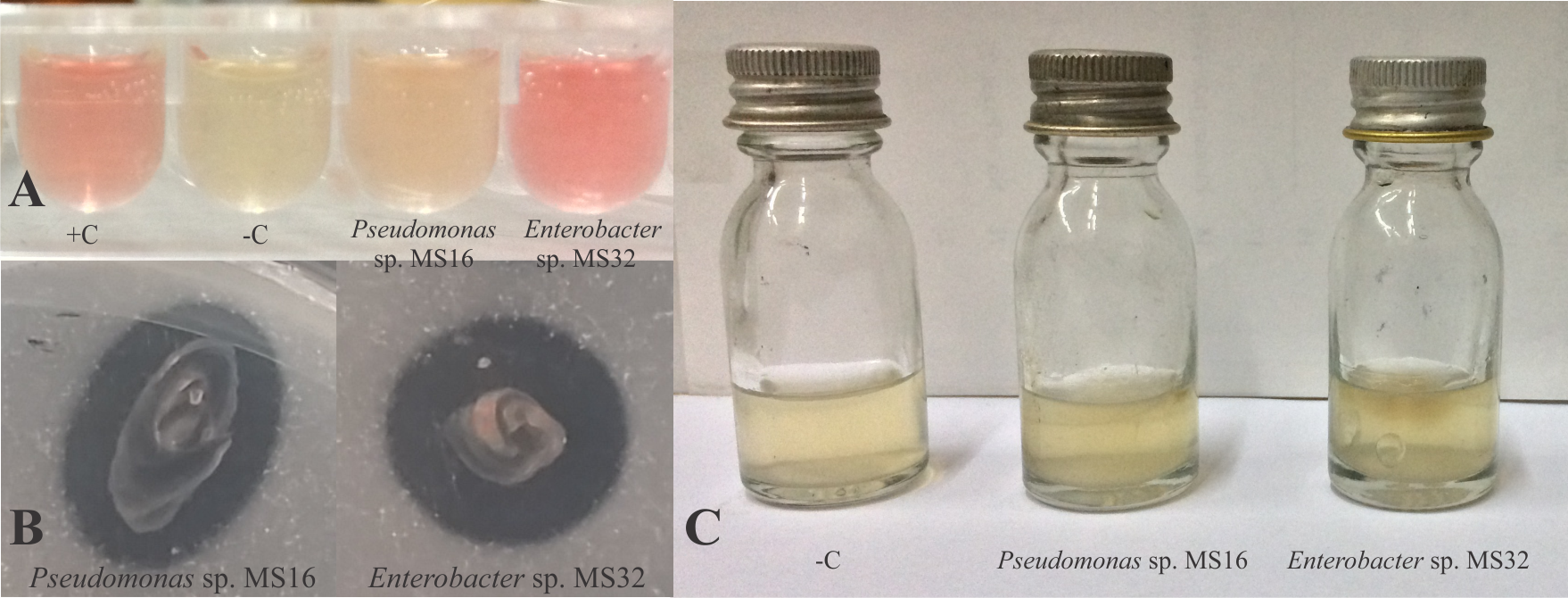

Supplement: S2 Fig — PGP traits of PSB was detected by IAA production as indicated by pink coloration upon reaction with Salkowski's reagent (A), Solubilization of zinc salts using tris minimal salt medium containing insoluble salts of zinc (B), and detection of ACC deaminase activity in vials containing 30 μL of 0.5 M ACC as a sole nitrogen source in 5 mL DF salt minimal medium (C). +C represent positive control while–C represent negative control (without inoculation). (TIF) [file pone.0204408.s002.tif]

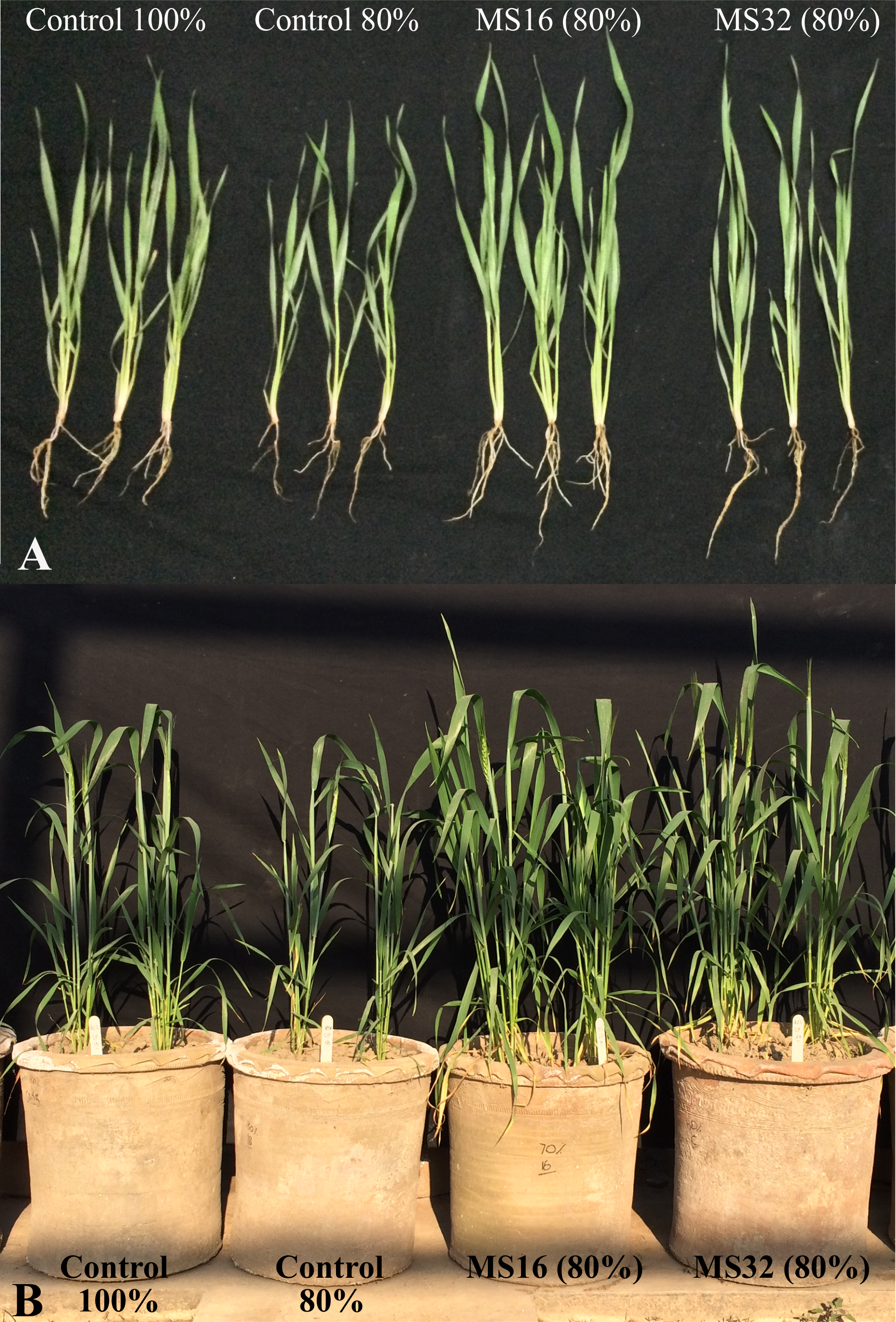

Supplement: S3 Fig — A: Root/ Shoot length at 35 days after sowing B: Wheat growth in pots inoculated with compared PSB as compared to un-inoculated controls. (TIF) [file pone.0204408.s003.tif]
