# Supplementary material for: Phosphate solubilizing bacteria with glucose dehydrogenase gene for phosphorus uptake and beneficial effects on wheat
Source: PLoS One. 2018 Sep 21;13(9):e0204408. doi: 10.1371/journal.pone.0204408 (PMC6150522; doi:10.1371/journal.pone.0204408)
Supplement: S1 Table — All information presented in this table are from current study. Soil was collected from experimental sites and analyzed for different physico-chemical properties. All values are an average of three biological replicates. (DOCX) [file pone.0204408.s004.docx]

S1 Table: Physico-chemical properties of soils collected from experimental field sites prior to sowing

| **Soil characteristics** | | **Experimental sites** | |
| --- | --- | --- | --- |
|  |  | **Faisalabad** | **Peshawar** |
| **Location** | Altitude | 596.3 feet | 998.4 feet |
|  | Latitude | 31°23'45.1"N | 34°00'48.2"N |
|  | Longitude | 73°01'34.2"E | 71°42'46.5"E |
| **Physico-chemical properties** | Sand (%) | 86 | 40 |
|  | Silt (%) | 12 | 28 |
|  | Clay (%) | 2 | 32 |
|  | Soil texture | Sandy Loam | Clay Loam |
|  | pH | 7.80 | 8.10 |
|  | Organic matter (%) | 0.85 | 0.67 |
| **Macronutrients** | Available P  (mg kg^-1^) | 1.90 | 1.30 |
|  | Total N (%) | 0.06 | 0.04 |
|  | Extractable K (mg kg^-1^) | 114 | 120 |

All information presented in this table are from current study. Soil was collected from experimental sites and analyzed for different physico-chemical properties. All values are an average of three biological replicates.
