# Supplementary material for: Phosphate solubilizing bacteria with glucose dehydrogenase gene for phosphorus uptake and beneficial effects on wheat
Source: PLoS One. 2018 Sep 21;13(9):e0204408. doi: 10.1371/journal.pone.0204408 (PMC6150522; doi:10.1371/journal.pone.0204408)
Supplement: S2 Table — Sequences of Strains were analyzed using NCBI GenBank database. (DOCX) [file pone.0204408.s005.docx]

S2 Table: The percentage homology of PSB 16S rRNA gene sequence with NCBI GenBank database

| **Strains** | **Organism Identified** | **Accession number** | **Closest type strain in NCBI Database** |
| --- | --- | --- | --- |
| MS14 | *Acenitobacter* | LT908021 | *Acenitobacterheamolatycus* |
| MS15 | *Pseudomonas* | LT908011 | *Pseudomonas cepacia* |
| MS16 | *Pseudomonas* | LT908012 | *Pseudomonas koreenisis* |
| MS18 | *Pseudomonas* | N/A | *Pseudomonas Putida* |
| MS26 | *Pseudomonas* | N/A | *Pseudomonas flourescens* |
| MS32 | *Enterobacter* | LT908013 | *Enterobacter cloacae* |
| MS47 | *Pseudomonas* | LT908014 | *Pseudomonas putida* |
| MS49 | *Pseudomonas* | LT908015 | *Pseudomonas putida* |
| MS52 | *Pseudomonas* | LT908016 | *Pseudomonas plecoglocicida* |
| MS101 | *Sinorhizobium* | LT908017 | *Sinorhizobium meliloti* |
| MS113 | *Acinetobacter* | LT908018 | *Acinetobacter calcoaceticus* |
| MS160 | *Lactococcus* | LT908047 | *Lactococcus lactis* |
| MS162 | *Acinetobacter* | LT908019 | *Acinetobacter calcoaceticus* |
| MS170 | *Pantoea* | LT908020 | *Pantoea agglomerans* |

Sequences of strains were analyzed using NCBI GenBank database. Strains were identified based on their maximum % homology, E-Value and query coverage.
