# Supplementary material for: Phosphate solubilizing bacteria with glucose dehydrogenase gene for phosphorus uptake and beneficial effects on wheat
Source: PLoS One. 2018 Sep 21;13(9):e0204408. doi: 10.1371/journal.pone.0204408 (PMC6150522; doi:10.1371/journal.pone.0204408)
Supplement: S3 Table — R2 = 0.9159 Adjusted R2 = 0.8212 Predicted R2 = 0.3584 Adequate precision = 7.909 * Significant at p<0.05. (DOCX) [file pone.0204408.s006.docx]

S3 Table: Analysis of variance for phosphate solubilization response (µg mL^-1^) using Response Surface Methodology

| **Source** | **Sum of squares** | **df** | **Mean Square** | **F Value** | **p-value** |
| --- | --- | --- | --- | --- | --- |
| Model | 483.26 | 9 | 53.70 | 6.16 | 0.0088* |
| Residual Error | 48.93 | 8 | 6.12 |  |  |
| Pure Error | 0.054 | 3 | 0.018 |  |  |

R^2^ = 0.9159

Adjusted R^2^ = 0.8212

Predicted R^2^ = 0.3584

Adequate precision= 7.909

* Significant at p<0.05
