# Supplementary material for: Phosphate solubilizing bacteria with glucose dehydrogenase gene for phosphorus uptake and beneficial effects on wheat
Source: PLoS One. 2018 Sep 21;13(9):e0204408. doi: 10.1371/journal.pone.0204408 (PMC6150522; doi:10.1371/journal.pone.0204408)
Supplement: S4 Table — Effect of PSB on soil nutrients in pots and microplots. Data is an average of three replicates. Data was taken at 120 Days after sowing (DAS). ± represents Standard Deviation. Means with significant difference (P<0.05) among treatments is represented by different letter. (DOCX) [file pone.0204408.s007.docx]

S4 Table: Effect of PSB inoculation on soil nutrients in pots and microplots

| **Site** | **Treatment** | **Soil available P (µg g^-1^ soil)** | **Soil Phosphatase Activity**  **(µmoles g^-1^ soil hr^-1^)** | **Soil Potassium Content**  **(µg g^-1^ soil)** | **Soil Sodium content**  **(µg g^-1^ soil)** |
| --- | --- | --- | --- | --- | --- |
| **Pots data** |  |  |  |  |  |
|  | *Pseudomonas* sp. MS16 | 2.72±0.10**a** | 22.55±0.43 **a** | 37.18±1.36**a** | 26.37±0.93**a** |
|  | *Enterobacter* sp. MS32 | 2.54±0.16**a** | 21.34±0.47 **b** | 34.93±0.48**ab** | 19.91±0.11**b** |
|  | Control 80% | 1.91±0.12 **b** | 18.47±0.24**c** | 30.29±1.36 **c** | 14.33±0.73**d** |
|  | Control 100% | 2.12±0.15 **b** | 17.84±0.28**c** | 34.32±1.78**b** | 17.25±0.92**c** |
| **Microplots**  **(2016-17)** |  |  |  |  |  |
|  | Inoculated | 2.78±0.08**a** | 23.22±0.40**a** | 40.95±1.95**a** | 26.91±0.46**a** |
|  | Control 80% | 1.27±0.16**c** | 16.55±0.40**c** | 26.45±1.89**b** | 14.33±0.33**b** |
|  | Control 100% | 2.38±0.09**b** | 19.32±0.45**b** | 27.69±1.17**b** | 14.61±0.08**b** |
| **Microplots**  **(2017-18)** |  |  |  |  |  |
|  | Inoculated | 3.20±0.06**a** | 25.08±0.42**a** | 43.68±2.43**a** | 28.52±0.61**a** |
|  | Control 80% | 2.01±0.17**c** | 19.98±0.54**c** | 30.68±2.53**c** | 17.09±0.80**b** |
|  | Control 100% | 2.73±0.13**b** | 23.13±0.33**b** | 36.40±1.25**b** | 17.50±0.41**b** |

Effect of PSB soil nutrients in pots and microplots. Data is an average of three replicates. Data was taken at 120 Days after sowing (DAS). ± represents Standard Deviation. Means with significant difference (P<0.05) among treatments is represented by different letter.
